# *devCellPy* is a machine learning-enabled pipeline for automated annotation of complex multilayered single-cell transcriptomic data

**DOI:** 10.1038/s41467-022-33045-x

**Published:** 2022-09-07

**Authors:** Francisco X. Galdos, Sidra Xu, William R. Goodyer, Lauren Duan, Yuhsin V. Huang, Soah Lee, Han Zhu, Carissa Lee, Nicholas Wei, Daniel Lee, Sean M. Wu

**Affiliations:** 1 Cardiovascular Institute, Stanford University School of Medicine, Stanford, CA USA; 2 Institute for Stem Cell Biology and Regenerative Medicine, Stanford University School of Medicine, Palo Alto, USA; 3 Division of Pediatric Cardiology, Department of Pediatrics, Stanford University School of Medicine, Palo Alto, USA; 4 Biopharmaceutical Convergence, School of Pharmacy, Sungkyunkwan University, Suwon, South Korea; 5 Division of Cardiovascular Medicine, Department of Medicine, Stanford University School of Medicine, Palo Alto, USA

**Keywords:** Stem-cell differentiation, Differentiation, Machine learning

## Abstract

A major informatic challenge in single cell RNA-sequencing analysis is the precise annotation of datasets where cells exhibit complex multilayered identities or transitory states. Here, we present *devCellPy* a highly accurate and precise machine learning-enabled tool that enables automated prediction of cell types across complex annotation hierarchies. To demonstrate the power of *devCellPy*, we construct a murine cardiac developmental atlas from published datasets encompassing 104,199 cells from E6.5-E16.5 and train *devCellPy* to generate a cardiac prediction algorithm. Using this algorithm, we observe a high prediction accuracy (>90%) across multiple layers of annotation and across de novo murine developmental data. Furthermore, we conduct a cross-species prediction of cardiomyocyte subtypes from in vitro*-*derived human induced pluripotent stem cells and unexpectedly uncover a predominance of left ventricular (LV) identity that we confirmed by an LV-specific TBX5 lineage tracing system. Together, our results show devCellPy to be a useful tool for automated cell prediction across complex cellular hierarchies, species, and experimental systems.

## Abstract (web summary)

A major informatic challenge in single cell RNA-sequencing analysis is the precise annotation of datasets where cells exhibit complex multilayered identities or transitory states. Here the authors present devCellPy, a Python-based package that enables the automated prediction of cell types across complex cellular hierarchies, species, and experimental systems with high accuracy, particularly for developmental scRNA-seq datasets.

## Introduction

Over the past decade, single-cell RNA sequencing (scRNA-seq) technologies have provided unprecedented insight into the transcriptional landscapes that regulate embryonic development, cell identities, and disease states^1–7^. As the number of cells obtained from these experiments continues to grow, bioinformaticians are faced with an increased burden to identify thousands of cells often through the laborious process of unsupervised clustering and manual cell type assignment^8^. Moreover, manual cell type assignment can result in high variability of cell annotation between research groups as well as poor reproducibility in cell identification between experiments^9^.

To address this challenge, multiple groups have developed informatics tools to assign cell identities that utilize reference datasets to map annotations onto newly collected data^10–16^. While these tools have provided powerful annotation algorithms, a major limitation is the lack of fully automated processes for classifying cells across complex hierarchies of annotations where cells exhibit multiple subclasses of identities or temporally restricted cell types. For example, in scRNA-seq data collected from developing embryos, cells exhibit dynamic and transitory cell states as well as finer cell identities. Certain cell identities may only be present during defined timepoints of development which creates an additional challenge when attempting to automatically assign cell identities using algorithms that do not account for temporal variables when training prediction models. Moreover, these finer identities are often undetectable without extensive sub-clustering of the data and recalculation of new dimensionally reduced feature spaces^1,9,17^. Automated cell prediction algorithms often require users to construct individual reference models to achieve annotation of highly granular subclasses of cells^11–13,15,17,18^. This challenge is especially highlighted in developmental datasets where cell types exist within restricted periods of development, therefore, complicating the generation of a unified prediction model that can assign cell identities based on the timepoints being queried.

To address these challenges, we present *Dev*elopmental *Cell* Prediction in *Py*thon (*devCellPy*) a Python-based package for the automated prediction of cell identities across highly complex annotation hierarchies obtained from any tissue or species. The basis for *devCellPy*’s prediction model is extreme gradient boosting (XGBoost), a supervised machine learning method that works by using a series of gradient-boosted decision tree ensembles to learn the set of input features needed to create accurate predictions^19^. Importantly, XGBoost has demonstrated resounding success in multiple fields ranging from medicine, finance, marketing, and public health and has previously been successful for cell classification through the CaSTLe algorithm implemented in R programming language^11,19^. Furthermore, we chose XGBoost due to its scalability for high dimensional data, computational efficiency, resistance to overfitting, and ability to handle missing values such as missing genes between datasets^1,2^. Moreover, XGBoost allows for automated identification and weighing of features used for making predictions which provides a direct understanding of how the algorithm makes certain classifications. *DevCellPy* provides a significant advance in the automated assignment of cell identities by learning the annotation hierarchy of a particular reference dataset and creating prediction models to classify cells across all layers of annotation in a fully automated manner. Importantly, the algorithm allows for timepoint variables to be incorporated into the annotation hierarchy, therefore, allowing for the classification of cell identities present within restricted time periods.

To demonstrate the power of *devCellPy* for multilayered cell prediction, we constructed a single large-scale cardiac developmental cell atlas from multiple publicly available scRNA-seq datasets spanning from E6.5 to E16.5 of murine heart development. We tested *devCellPy* on a cardiac developmental dataset due to the highly complex number of distinct cell type annotations present during the development of the heart. The heart is derived from two populations of highly similar progenitor populations known as the first and second heart fields which give rise to distinct cardiac regions^1,20–22^. Importantly, cell types within the heart exhibit anatomically patterned gene expression patterns that vary through time, thus providing an optimal system to illustrate the predictive power of *devCellPy*^1,22^.

We trained *devCellPy* on this cardiac developmental atlas and validated the algorithm’s highly accurate (>90%) predictive capacity across multiple layers including time-restricted cell populations. Moreover, we successfully applied our *devCellPy-*generated cardiac prediction algorithm on data unseen during training and demonstrated its accuracy relative to previously published cell prediction algorithms. Lastly, we applied our algorithm to predict cardiac differentiation outcomes of human-induced pluripotent stem cells (hiPSCs) and found, surprisingly, a left ventricular cardiomyocyte predominant differentiation of hiPSCs. This result was further validated using an in vitro lineage tracing strategy with a *TBX5*-*Cre*/LoxP and *MYL2* fluorescence reporter, demonstrating the broad applicability of our algorithm for predicting cardiac cell types across species.

## Results

### *devCellPy* enables the generation of a multilayered prediction algorithm for cell type and subtype classification

**Fig. 1:**
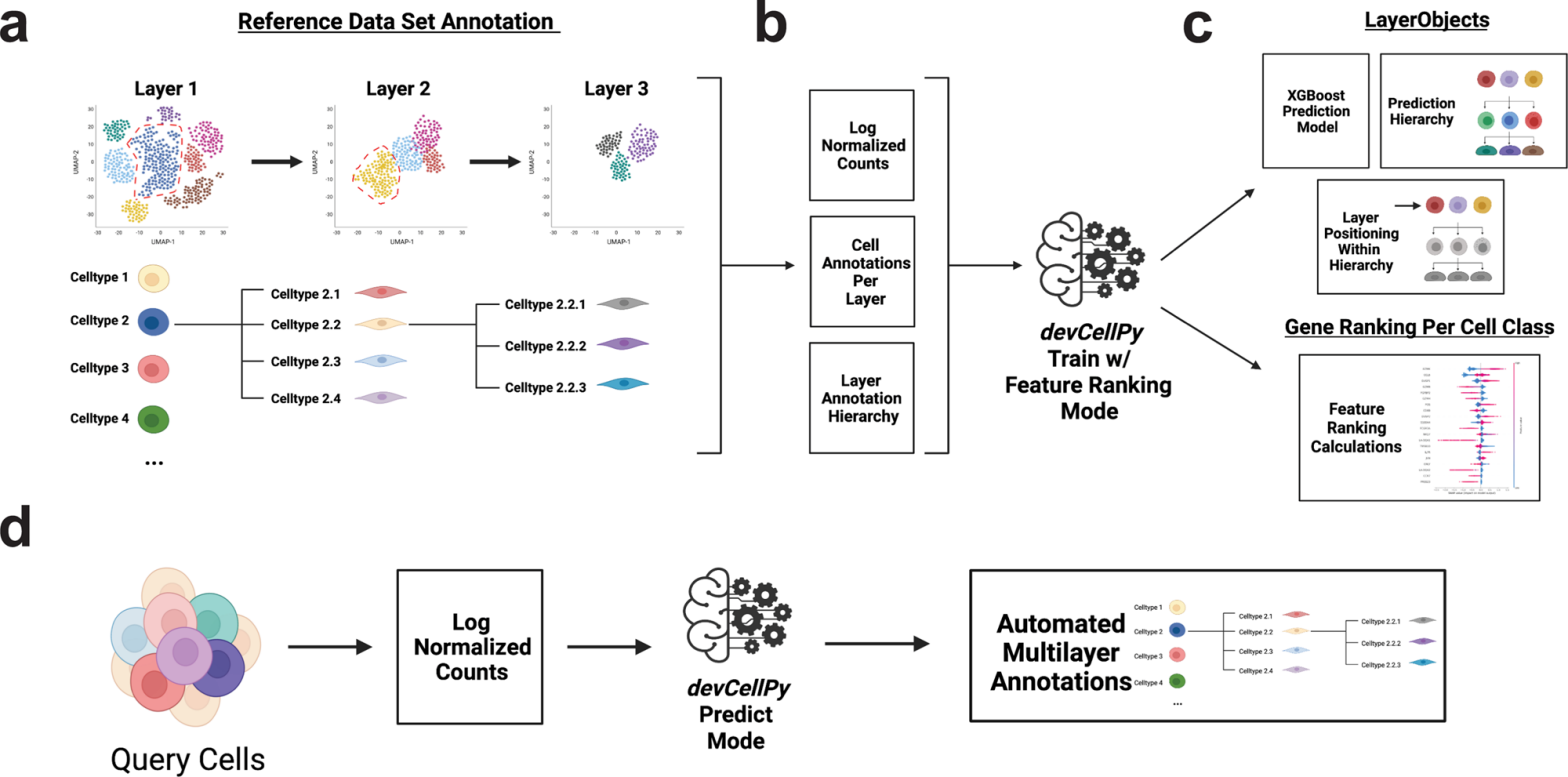
Overview of *devCellPy*. *devCellPy* is a multilayered machine learning algorithm for the hierarchical annotation of single-cell RNA-seq data. **a** Reference data is constructed by conducting annotation of the dataset across multiple layers. These layers follow a hierarchical structure where cell subtype annotations are nested within broader categories. The hierarchical structure can be present for any combination of layers and sublayers. **b** To train *devCellPy*, log normalized counts, individual cell annotations per layer, and a layer annotation hierarchy are fed into the algorithm’s train mode. **c** *devCellPy* will create a LayerObject for each layer of annotation within the hierarchy. The LayerObject consists of an XGBoost prediction model that is trained on the reference data and will also encode the position of the layer within the annotation hierarchy. LayerObjects allow for the automated prediction of cell types across all layers of annotation. Importantly, *devCellPy* follows the hierarchy’s organizational logic meaning that cell subtypes will be predicted only if they fall within a specified branch within the hierarchy. Under the feature ranking mode, *devCellPy* will use the SHAP algorithm to determine the positive and negative gene predictors per cell type annotated. **d** *devCellPy* can conduct automated prediction of query cells by exporting log normalized counts and feeding the matrix to the algorithm. The output will contain an automated annotation across multiple layers.

To address the challenge of automated classification of cell types and subtypes in a hierarchical fashion, we constructed *devCellPy*, a Python-based package for the generation of a cell identity prediction algorithm that incorporates the element of time. *devCellP*y consists of training and prediction steps. During the training component, an annotated reference dataset containing multiple layers of annotation is used for training the algorithm (Fig. 1a and in the section “Methods”). Users provide *devCellPy* with an annotation hierarchy specifying multiple layers and classes of cells within the dataset, including timepoint variables to construct an annotation hierarchy that is cell type and time-dependent. Furthermore, users provide a log-normalized expression matrix for all cells in the reference data and a metadata table containing single cell annotations across all layers of the hierarchy (Fig. 1b). We introduce the LayerObject class within *devCellPy* to create an organized data structure where the algorithm learns the annotation hierarchy of a dataset and contains information of each layer’s position within the hierarchy (Fig. 1c). This system allows for the automated classification of cell subtypes across the correct branches of the hierarchy. An XGBoost prediction model is trained for each layer of the hierarchy and is stored within its layer’s respective LayerObject.

In addition to LayerObjects, we also implemented the recently developed Shapley Additive exPlanations (SHAP) algorithm into *devCellPy*^23^. SHAP is an algorithm that allows for the identification of the features used by tree ensemble methods to make predictions^23^. This method allows *devCellPy* to output gene markers that are automatically identified during training for making cell type classifications thus highlighting the top positive and negative gene markers used for classifying cell types in a dataset of interest (Fig. 1c). After training *devCellPy* on a reference dataset, users can then use the *devCellPy*-generated prediction algorithms to classify a query dataset by exporting a log normalized counts matrix that is loaded directly into the algorithm (Fig. 1d). *devCellPy* will automatically read in the matrix file and will use the prediction models stored in the LayerObjects to output a multilayered cell type and subtype prediction of the query cells. In addition to providing output annotations, *devCellPy* will also output probability metrics for each cell classified to provide users with information on the confidence of the algorithm in making cell predictions (see the “Methods” section). Overall, the algorithm is structured to allow users to create trained models based on any reference scRNA-seq dataset acquired from any tissue from any model organism and can easily export these models to conduct predictions on new datasets of similar cell types and subtypes.

### Construction of large-scale cardiac developmental atlas

**Fig. 2:**
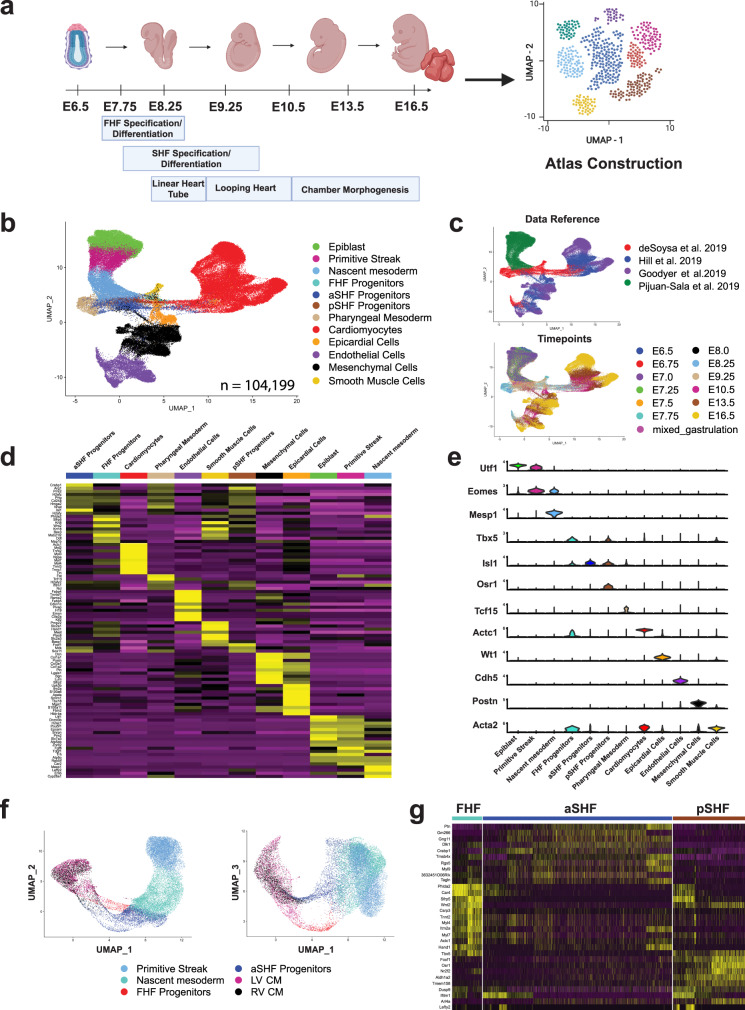
Compilation and annotation of mesoderm-derived cardiac developmental cell atlas. Data from four previously published datasets were downloaded, annotated, and integrated to construct a cardiac developmental cell atlas of mesoderm-derived cell types. **a** Schematic of major timepoints is included in the compiled atlas along with labeling of major events during cardiac development. **b** UMAP plot showing integration and batch correction of all annotated datasets into a unified developmental trajectory. **c** UMAP plots demonstrating the labeling and positioning of cells from all four reference datasets and timepoints included in the cardiac atlas. **d** Top 10 differentially expressed markers averaged across all cell types and presented in a heatmap plot. **e** Violin plot representation of top 12 markers most highly expressed markers across all major cell types. **f** UMAP plots displaying three-dimensional UMAP axes showcasing first heart field (FHF) and anterior second heart field (aSHF) branching trajectories from mesodermal progenitors into left (LV) and right (RV) ventricular cardiomyocytes, respectively. **g** Top differentially expressed markers across three major cardiac progenitors identified during assembly of the cardiac atlas.

To test the performance of *devCellPy* at generating highly accurate prediction algorithms for conducting multilayered cell annotations, we first assembled a large-scale scRNA-seq atlas of mesoderm-derived cardiac developmental cell types from four publicly available datasets^24–26^. To construct this atlas from independent datasets, we downloaded raw data from three major studies profiling developing embryonic mouse hearts spanning from cardiac progenitors to late-stage cardiac maturation stages of development (Fig. 2a). Each individual dataset underwent a first layer annotation of unsupervised clusters (Supplementary Fig. 1). After identifying major mesoderm-derived cell populations relevant to cardiac development^20,21,27^ (Fig. 2b, Supplementary Figs. 1, 2), we integrated the data from these three cardiac datasets with early cell types from a recently published gastrulation cell atlas^2^. Using the mutual nearest neighbor correction algorithm^28^, we integrated all datasets and observed the emergence of a clear developmental trajectory spanning from early gastrulation to terminal differentiation of major cardiac cell types encompassing a total of 104,199 cells (Fig. 2b, c).

By focusing on mesoderm-derived cell types, we observed a differentiation tree emerging from pluripotent epiblast cells followed by the transition through the primitive streak into early nascent mesodermal progenitors and progressing into cardiac progenitors (Fig. 2b). Despite these datasets originating from four distinct sources, we were able to successfully integrate all datasets and observe clustering of 12 major cell types across developmental time (Fig. 2b, c). The UMAP plot displayed a developmentally consistent structure with cardiac progenitors composing the trunk of the tree and subsequently multifurcating into distinct cell types such as cardiomyocytes, endothelial cells, epicardial cells, and immature smooth muscle cells as has been previously described^21,29–31^. We further validated the annotations assigned in the construction of the atlas by conducting differential gene expression analysis across all major annotated cell types and confirmed the unique expression of major cell markers that have been reported for the 12 major cell populations identified (Fig. 2d, e, Supplementary Figs. 1, 2, Supplementary Data 1). Among the first layer of annotation, we also confirmed the identity of three major populations of cardiac progenitors known as the first heart field (FHF), anterior SHF (aSHF), and posterior SHF (pSHF) progenitors that give rise to distinct anatomical regions of the heart^24,32–34^ (Fig. 2f, g). We identified distinct populations of major cell types in the developing heart including mesenchymal cells, endothelial cells, epicardial cells, and a small population of immature smooth muscle cells that displayed positive expression for late smooth muscle marker *Acta2*, and expression of multiple transcriptional regulators reported to regulate smooth muscle contractile machinery^35^ (*Foxf1)* and early outflow tract smooth muscle development^36^ (*Hand1* and *Twist1)* (Supplementary Data 1). Importantly, we identified the distinct lineage trajectory of the FHF and aSHF in giving rise to the left and right ventricles, respectively^21,24,34,37^ (Fig. 2f, g).

**Fig. 3:**
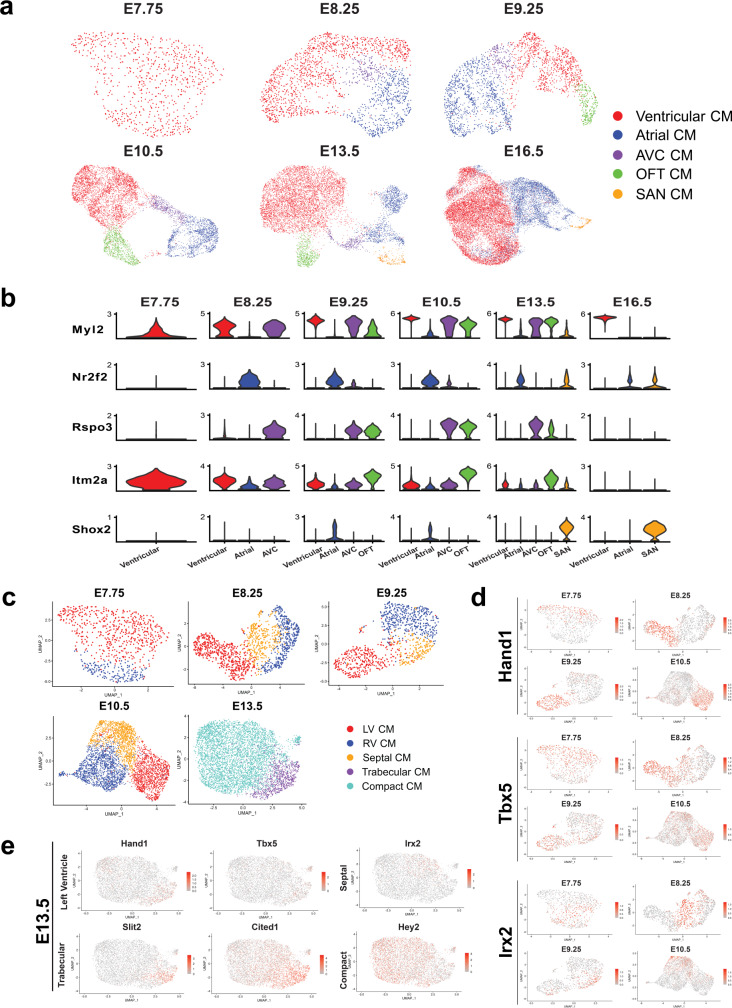
Annotation of cardiomyocyte and ventricular cardiomyocyte subtypes across multiple developmental timepoints. Cardiomyocytes identified during general cell type annotation were further annotated into distinct anatomical zones present during cardiac development. **a** UMAP plots and annotations for cardiomyocyte subtypes across all timepoints included in the cell atlas. Cell numbers per timepoints: E7.75 = 737, E8.25 = 2002, E9.25 = 2513, E10.5 = 7186, E13.5 = 8226, E16.5 = 18243. **b** Violin plots illustrating expression of five major markers specifically expressed across ventricular, atrial, atrioventricular canal (AVC), outflow tract (OFT), and sinoatrial node (SAN) cardiomyocytes. **c** UMAP plots for finer clustering and annotation of ventricular cardiomyocytes only. Clusters are labeled according to left (LV), right (RV), septal, trabecular, and compact cardiomyocyte identities. Cell number per timepoint: E7.75 = 737, E8.25 = 1284, E9.25 = 941, E10.5 = 4025, E13.5 = 5560. **d** Feature plots indicating enrichment of LV-specific markers (*Hand1, Tbx5)* and septal marker, *Irx2*, in respective annotated clusters. **e** Feature plots indicating the absence of early LV and septal markers by E13.5 and the expression of trabecular (*Slit2, Cited1)* and compact (*Hey2)* markers.

Due to the transcriptional similarity between cardiomyocyte (CM) subtypes, we were unable to distinguish these cells when clustered with non-cardiomyocytes (Fig. 2b). We, therefore, conducted sub-clustering of cardiomyocytes only, which revealed multiple cardiomyocyte subtypes including a ventricular, atrial, atrioventricular canal (AVC), outflow tract (OFT), and sinoatrial node cardiomyocytes (SAN) (Fig. 3a). Given the availability of anatomically annotated scRNA-seq atlases that we and others have published for mouse embryonic cardiomyocytes^1,22,25^, we were able to use validated markers to accurately annotate each cardiomyocyte subtype^1,22,24,25^. Within each timepoint, we found that specific sets of markers such as *Myl2, Nr2f2, Rspo3, Itm2a*, and *Shox2* consistently identified specific cardiomyocyte subtypes (Fig. 3b). Despite the lack of anatomical information provided by the source data, we were able to accurately reconstruct cardiomyocyte subtypes by registering each cell cluster to the genes that correspond to distinct anatomical regions of the heart.

To further refine the cellular resolution of our cardiac reference atlas, we assessed whether subtypes of ventricular cardiomyocytes (e.g. left, right, and septal) could be distinguished by incorporating an additional layer of annotation specifically within the ventricular cardiomyocyte subpopulation. We relied on the well-known expression of LV markers (e.g. *Tbx5* and *Hand1*) to annotate LV CMs between E7.75–E10.5^1,22,24,33,38^. RV CMs, on the other hand, showed an absence of expression of these two genes (Fig. 3d and Supplementary Data 1) consistent with prior reports^1,22,33,38,39^. At E8.25 we observed three distinct clusters including one cluster that highly expressed Iroquoix Homeobox 2 (*Irx2*), a transcription factor previously reported to be expressed in the developing interventricular septum (Fig. 3d and Supplementary Data 1)^40^. We identified additional clusters of *Irx2* expressing ventricular cardiomyocytes at E9.25 and E10.5 suggesting that an early transcriptional signature of septal cells could be detected during the initial heart looping stages of cardiac development and the emergence of a primordial interventricular septum^40,41^. By E13.5, the differences between LV, RV, and Septal CMs could no longer be discerned, however, we were able to identify trabecular and compact cardiomyocytes consistent with the process of trabeculation at this stage (Fig. 3e). By E16.5, differences between left and right ventricular cardiomyocytes could no longer be clearly delineated based on established LV/RV/Septal markers likely due to the high degree of transcriptional similarity between these ventricular cardiomyocyte subpopulations at later stages of development (Supplementary Fig. 3).

### *devCellPy-*generated algorithm accurately predicts cell types across a complex hierarchy of annotation layers

**Fig. 4:**
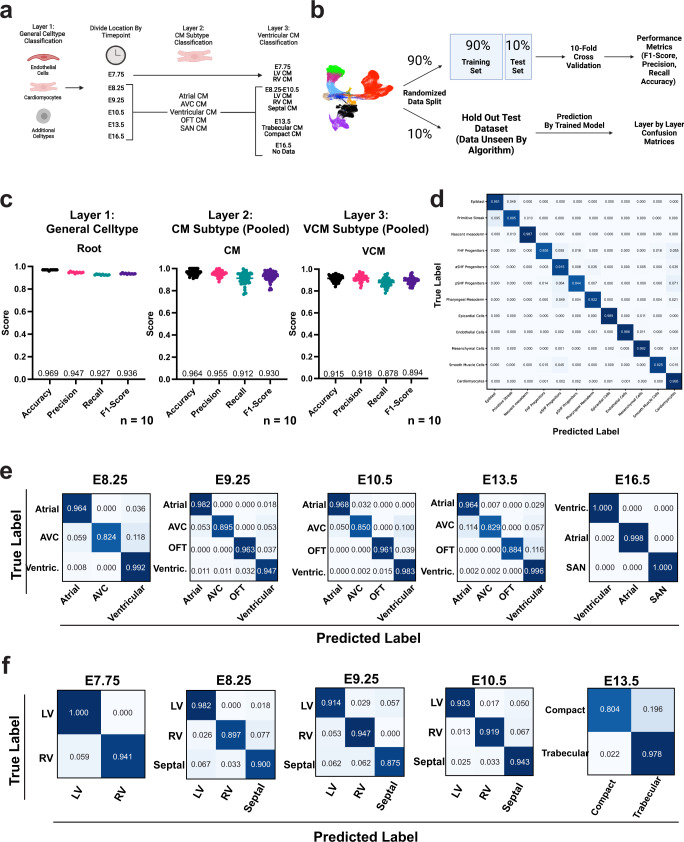
*devCellPy* shows high classification performance metrics across individual layers during cross-validation. *devCellPy* was tested on the compiled cardiac developmental cell atlas to determine the classification performance of the algorithm. **a** Annotation scheme demonstrating the hierarchical annotation layout of the cardiac developmental atlas. **b** Schematic of the training and testing workflow for the evaluation of *devCellPy* across all layers of annotation. Log normalized counts matrices containing all cells were randomly split into a 90% partition used for 10-fold cross-validation and a 10% partition hold-out dataset used for final testing of the model’s accuracy. **c** Performance metrics calculated for 10-fold cross-validations for each layer of annotation. Layers 2 and 3 displayed pooled results for all timepoints. Error bars represent 95% confidence intervals. *N* = 10 for independent folds were tested. **d** Confusion matrix for layer 1 of annotation indicating classification accuracies across general cell types. **e** Confusion matrices for layer 2 representing cardiomyocyte subtypes across distinct timepoints. **f** Serial confusion matrices for layer 3 ventricular cardiomyocyte subtypes between E7.75–E13.5 of murine development.

Having established a large cardiac developmental atlas containing multiple layers of annotation, we proceeded to test the ability of *devCellPy* to generate a highly accurate cell identity prediction algorithm on this dataset. The cardiac cell atlas was analyzed in multiple layers including a top layer representing broad cell annotations, followed by cardiomyocyte subtypes, and ventricular cardiomyocyte subtypes divided by timepoints during development (Fig. 4a). To test the classification performance of the algorithm, we randomly partitioned the data into a 90% and 10% partition that was used for cross-validation and hold-out dataset testing, respectively (Fig. 4b). The 90% partition was used for 10-fold cross-validation by retraining the model 10 independent times and tested on a partition of data that was randomly stratified from each class of cells (Fig. 4b, see the section “Methods”). We evaluated four major performance metrics including the accuracy of the model, precision, recall, and the F1-score (Fig. 4c–e, Supplementary Data 2). For the first layer of annotation, the model displayed high overall accuracy 96.9 ± 0.2% across 10 independent rounds of training (Fig. 4c). We also observed the model was highly sensitive given a recall value of 92.7 ± 0.30% and displayed an overall precision score of 94.7 ± 0.4% (Fig. 4c). *devCellPy’s* classification of the 10% held-out data, unseen by the trained model, confirmed highly accurate predictions with 11 out of 11 cell classes of the first annotation layer predicted with a >84% accuracy and classes such as cardiomyocytes and mesenchymal cells displaying >99% prediction accuracy (Fig. 4d).

We next evaluated the performance of the *devCellPy-*generated cardiac algorithm in classifying cardiomyocyte and ventricular cardiomyocyte subtypes. Consistent with our first layer results, we observed a mean accuracy of 96.4 ± 0.60% when predicting distinct classes of cardiomyocytes across distinct timepoints during development (Fig. 4c and Supplementary Fig. 4A), as well as high precision (95.5 ± 0.7%), and recall (91.2 ± 1.5%) scores. Interestingly, the slight decrease in model recall was likely due to the decrease in calculated accuracy between cell types such as the AVC, OFT, and SAN CMs. Closer inspection of the confusion matrix calculated on the hold-out data shows that the AVC and OFT CM displayed misclassification with ventricular CMs which closely border these cells anatomically (Fig. 4e). Interestingly, no misclassification between AVC and OFT CM was observed, indicating that the *devCellPy-*generated algorithm misclassified only between anatomically adjacent cardiomyocyte subtypes.

Further analysis of the cardiac cell prediction algorithm performance of ventricular cardiomyocyte subsets further revealed that across all developmental timepoints between E7.75–E13.5, the pooled average accuracy was 91.5 ± 0.8% (Fig. 4c and Supplementary Fig. 4B). As observed with cardiomyocyte subtypes, confusion matrices at each timepoint displayed high overall accuracy across each ventricular cardiomyocyte subtype analyzed. Interestingly, we observed that septal cardiomyocytes displayed the lowest accuracy score with the greatest confusion between LV and RV CMs (Fig. 4f). This was expected given that the septum borders these two cardiac chambers. Overall, we observed that performance metrics exhibited the highest scores at the first layer annotation with slight decreases within closely related cell types found within the lower levels of the annotation hierarchy.

To further evaluate the performance accuracy of *devCellPy*, we compared the performance of the algorithm to previously published single-cell prediction algorithms using a subset of cells from the well-cited *Tabula muris* dataset^42^ (Supplementary Fig. 5A). We selected 16 cell types from this *Tabula muris* to conduct this evaluation and found that across a 10-fold cross-validation, *devCellPy* was statistically more accurate (*p*-value < 0.0001) than previously published single-cell prediction algorithms CaSTLE^11^, SeuratV3, scmap^18^, or SingleCellNet^43^, while being non-significantly different from scPred^13^ (Supplementary Fig. 5B). Moreover, we conducted a test on the same 10% hold out dataset for all classification algorithms to evaluate overall accuracy across individual cell types. Overall, *devCellPy* displayed the highest overall accuracy across all cell types evaluated, with other algorithms displaying <90% accuracy across multiple cell type categories. Of note, CaSTLE, another XGBoost-based package displayed multiple cell types with <85% accuracy. In addition to displaying a high performance relative to previously published algorithms, we also found that the minimum number of cells to achieve >95% overall accuracy for *devCellPy* was 128 cells per cell type with accuracies of 97% reached at 512 cells per cell type (Supplementary Fig. 6A–C). Importantly, even when *devCellPy* was trained on as low as 16 cells per cell type, the algorithm continued to show accuracies above 98% across multiple cell types with only three cell categories (macrophage, stromal cell, and blood cell) falling below 90%.

### *devCellPy* identifies biologically relevant cell markers

We asked whether the *devCellPy-*generated cardiac prediction algorithm could identify developmentally relevant genes for making predictions across cell layers. Using the SHAP algorithm within *devCellPy*^23^, we identified the top positive and negative predictor genes for cell types across each layer of annotation tested (Supplementary Figs. 7 and 8). For layer 1, the algorithm automatically identified previously validated predictors of cell identity (Supplementary Fig. 7). For example, top markers identified included well-known cardiac sarcomere markers of cardiac muscle identity including *Tnnt2, Actc1, Ttn*, and *Actn2*^1,22,24,44,45^. Similarly, top-ranking features for endothelial cells included well-known markers *Cdh5* and *Pecam1*^46,47^, while *Postn* and *Col3a1* were identified as top mesenchymal cell markers^48^. Interestingly general cell type predictors revealed major positive predictors of known cell types such as *Utf1* in epiblast cells^49^, *Mesp1* in nascent mesoderm^27,50^, *Isl1* in anterior second heart field progenitors^37^, and *Osr1* in posterior second heart field progenitors^32^ (Supplementary Fig. 4). In addition to identifying canonical markers for general cell types, SHAP identified additional markers for cell types such as FHF Progenitors that have only recently been uncovered as markers for these progenitors. *Mab21l2* was a top positive predictor of FHF Progenitors which has recently been described as a marker of a subset of FHF progenitors giving rise to LV and epicardial cells^31,51^. Interestingly, we did not observe canonical FHF markers such as *Tbx5* and *Hand1* as top-ranking markers used by the algorithm likely due to the expression of these markers in other general cell types such as cardiomyocytes. Across all major cell types, top-ranking markers were consistent with known markers in the literature and did not exhibit a high number of housekeeping or ribosomal genes due to batch-specific technical noise.

In addition to identifying top markers of general cell types in layer 1, we determined top-ranking genes for the identification of cardiomyocyte and ventricular cardiomyocyte subtypes across multiple timepoints of differentiation (Supplementary Fig. 8). Interestingly, *devCellPy* identified multiple previously published markers for cardiomyocyte subtypes across time, while also identifying unique markers in a timepoint specific manner. For atrial CMs, the top positive predictors included genes such as *Nr2f1*, *Nr2f2*, and *Stard10*, while for ventricular CM positive predictors included genes such as *Myl2*, *Irx4*, and *Myh7* (Supplementary Fig. 8)^1,22,52,53^. Similarly, for OFT CM and AVC CM, top ranking genes included *Rspo3* for identifying both cell types, *Itm2a* as a positive predictor for OFT CM, and *Tbx3* for AVC (Supplementary Fig. 8). All three genes have been identified as top markers for identifying OFT and AVC CM in previous anatomical profiling of E10.5 murine hearts^1^. SHAP ranking also identified unique expression markers expressed in the sinoatrial node pacemaker cells including *Shox2, Igfbp5*, and *Smoc2* (Supplementary Fig. 8)^25,54,55^.

SHAP analysis also identified multiple top candidate markers for LV, RV, and Septal ventricular cardiomyocytes. Top LV predictors included genes such as *Wnt2* for E7.75 cardiomyocytes (Supplementary Fig. 9A), which has been recently cited as an early secreted factor of the first heart field and early heart tube stage LV cardiomyocytes^56^. Consistent with the dynamic changes that occur during development, the markers present during each timepoint varied. However, well-established markers for LV such as *Hand1* ranked as the top-ranking gene from E8.25-E10.5 (Supplementary Fig. 9A). For RV predictors, reciprocal patterns were observed with LV markers such as *Hand1* at E9.25–E10.5, while also showing expression of *3632451O06Rik* (Supplementary Fig. 9B), which has been reported to be expressed in an RV specific pattern in developing mouse hearts^24^. Interestingly, the primary septal predictors (Supplementary Fig. 9C) included previously reported markers including *Irx1* and *Irx2*^40,41^. Due to the lack of distinct gene expression differences between LV/RV/septal cardiomyocytes beyond E13.5, we focused on trabecular and compact cardiomyocyte differences and confirmed that the *devCellPy-*generated cardiac prediction algorithm identified known markers for trabecular CMs *Mest* and *Nppa* (Supplementary Fig. 9D).

**Fig. 5:**
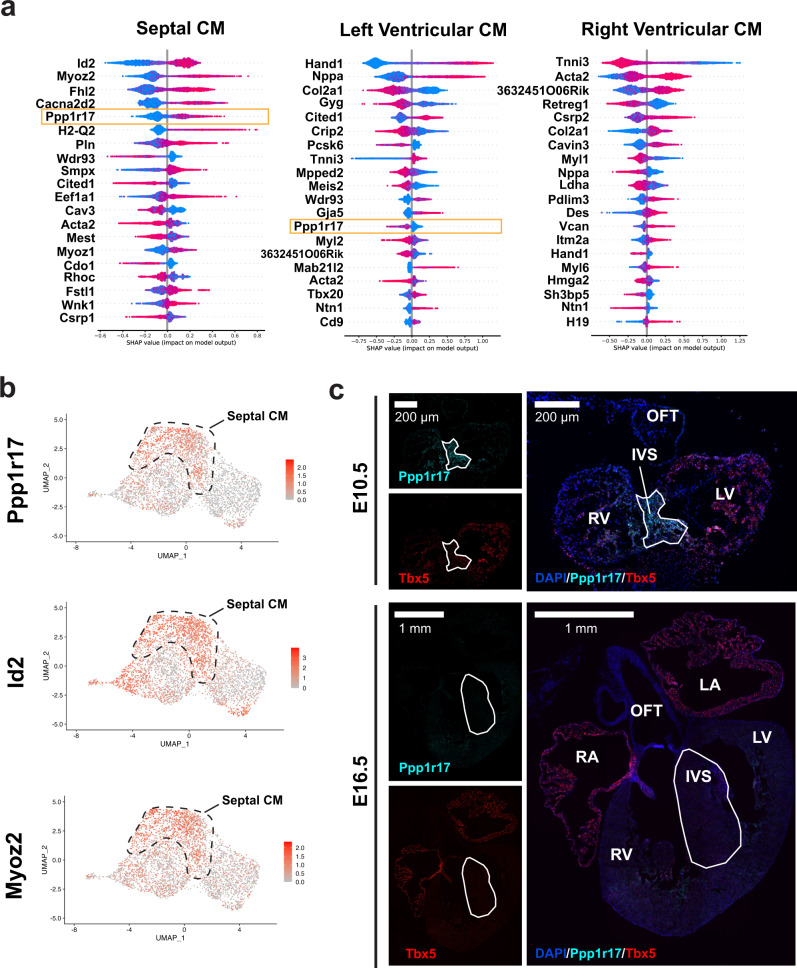
Identification of Septal Marker *Ppp1r17* by *devCellPy*. *devCellPy* feature ranking using the SHAP algorithm identified markers of septal cardiomyocyte identity. **a** Top positive and negative predictors of ventricular cardiomyocyte identity at E10.5. Orange box indicates the identification of *Ppp1r17* as a septal marker. **b** Feature plots indicating the expression of three putative septal markers identified by SHAP. **c** Representative RNAScope images for *Ppp1r17* (cyan), *Tbx5* (red), DAPI (blue) at E10.5 and E16.5 of murine development. A minimum of three biological (different hearts) and three technical (different sections/heart) were used for each in situ hybridization with similar results to the representative image presented. Scale bars are 200 µm and 1 mm, respectively.

Given that SHAP identified multiple previously confirmed and putative markers for cardiomyocyte subtypes, we probed by RNAScope one of the previously unreported markers identified by SHAP as a high-ranking positive predictor of septal CMs. At E10.5, we identified multiple potential candidate septal markers (Fig. 5a). By single-cell RNA sequencing, we identified *Ppp1r17*, *Id2*, and *Myoz2* to display a septal-specific pattern (Fig. 5b). Using RNAScope, we validated *Ppp1r17* as a marker of the interventricular septum, showing exquisite specificity at E10.5 when probed alongside LV marker *Tbx5* (Fig. 5c). Interestingly, labeling at E16.5 revealed that both *Ppp1r17* and *Tbx5* both exhibit a septal and LV specific expression pattern during early development, which is lost by later stages of development (Fig. 5c). Overall, these findings present *devCellPy* as a powerful tool for identifying candidate genetic markers across developmental time.

### *devCellPy*-generated algorithm accurately predicts cell types from de novo datasets

**Fig. 6:**
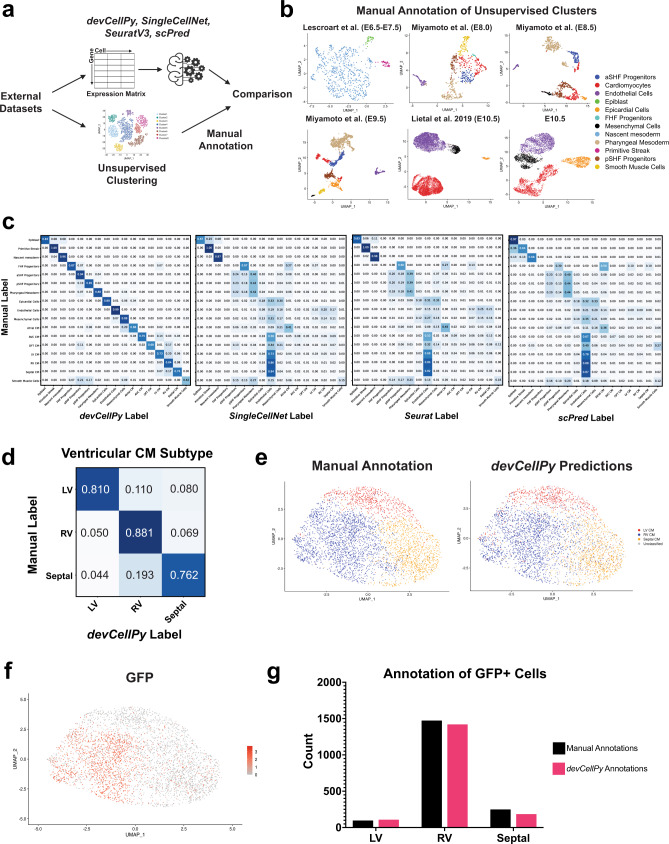
*devCellPy* prediction of new scRNA-seq data and *Isl1*-lineage traced ventricular cardiomyocytes. Single cells from three previously published cardiac datasets and freshly collected E10.5 hearts were manually annotated and run through the *devCellPy* prediction algorithm. **a** Schematic of workflow for evaluation of manual versus *devCellPy* prediction of cell types. Datasets that *devCellPy* has not previously encountered (external datasets) were either analyzed by manual unsupervised clustering or normalized expression matrices fed directly into the *devCellPy* algorithm for cell type classification. **b** UMAP plots with manual annotations for layer 1 (general cell types) unsupervised clusters across individual timepoints evaluated. **c** Confusion matrix representations comparing manual annotations with the classification labels generated by *devCellPy, SingleCellNet, Seurat, and scPred*. Diagonal represents percentage of labels that agree between both classification methods. **d** Confusion matrix comparing LV, RV, and Septal classifications between manual and *devCellPy* for Li et al. 2019 ventricular cardiomyocytes. **e** UMAP plots with annotation labels for manual and *devCellPy* predictions of ventricular cardiomyocytes from Li et al. 2019 dataset. **f** Feature plot showing expression of GFP in single cells from the hearts of *Isl1*-Cre/Rosa26-mTmG mice (Li et al. 2019 dataset). **g** Quantification of GFP-positive cells classified as LV, RV, and septal cardiomyocytes.

To further evaluate the performance of *devCellPy*, we asked whether the *devCellPy-*generated prediction algorithm could successfully predict cell types in datasets it had not previously encountered. We analyzed scRNA-seq data from three new sources^50,56,57^ as well as generated our own data from E10.5 mouse hearts and tested *devCellPy’s* ability to perform a fully automated prediction of cardiac cell types across all cell types present in the cardiac atlas. Importantly, we further evaluated the ability of other cell prediction algorithms to conduct the full classification of all cell types in the cardiac atlas (Fig. 6a, b and Supplementary Fig. 10). We compared the machine learning classifications to the manual annotations assigned during unsupervised cluster annotation of the query datasets (Fig. 6b and Supplementary Fig. 10). Comparison of manual annotations and *devCellPy*’s predictions revealed a high degree of concordance between the two annotation methods with a >80% accuracy across 11 out of 17 cell categories (Fig. 6c). Among the categories that reported below 90% agreement between *devCellPy* and the manual method were included major cardiomyocyte subtypes and ventricular cardiomyocyte subtypes that exhibited >70% concordance, with the exception of atrial cardiomyocytes.

In comparison to other previously published machine learning methods, *devCellPy* outperformed *SingleCellNet, Seurat*, and *scPred* in cell classification across all cell types present in the cardiac atlas. Interestingly, all other methods displayed high concordance with the manual method for Epiblast, Primitive Streak, and Nascent Mesoderm, but observed low prediction accuracies across all other cell types. Interestingly, across closely related cell types such as cardiac progenitors, epicardial and mesenchymal cells, and cardiomyocyte subtypes other methods showed low concordance between the manual method and the machine learning predictions (Fig. 6c). Moreover, we observed nearly all other methods misclassified cardiomyocyte subtypes.

To further determine the accuracy of *devCellPy*, we used the Li et al. dataset that contained an *Isl1*-driven Cre/LoxP lineage tracing marker for labeling RV cardiomyocytes as a way to validate *devCellPy’s* accuracy in identifying this *Isl1* lineage-labeled cells^57^. Comparison between the manual and *devCellPy* methods showed >75% concordance between the two methods (Fig. 6d, e). Reassuringly, the cluster that was annotated as RV in both manual and *devCellPy* annotation was also predominantly GFP-positive (Fig. 6f, g). Given that the cardiac cell prediction algorithm generated by *devCellPy* was trained on data that did not contain GFP, we were able to validate the accuracy of the algorithm by observing that most GFP-positive cells were classified as RV (Fig. 6g). Interestingly, a comparison of both *devCellPy* and manual annotations revealed almost identical numbers of GFP+ cells being predicted as RV, with 1451 GFP+ cells predicted as RV in the manual method and 1398 in the *devCellPy* method. While we detect a low number of GFP+ cells in the LV classified cells, this was present for both methods and likely represented small numbers of cells in the left ventricle expressing GFP which was also observed in the original publication^57^. Overall, these results provide multiple lines of evidence that a *devCellPy-*generated prediction algorithm can accurately predict cell types in de novo datasets and outperform other methods when conducting predictions on highly granular cell categories such as cardiac progenitors and multiple subclasses of cardiomyocytes.

### *devCellPy-generated* cardiac prediction algorithm reveals developmental immaturity and ventricular-specific cardiomyocyte differentiation of hiPSC-derived cardiomyocytes

**Fig. 7:**
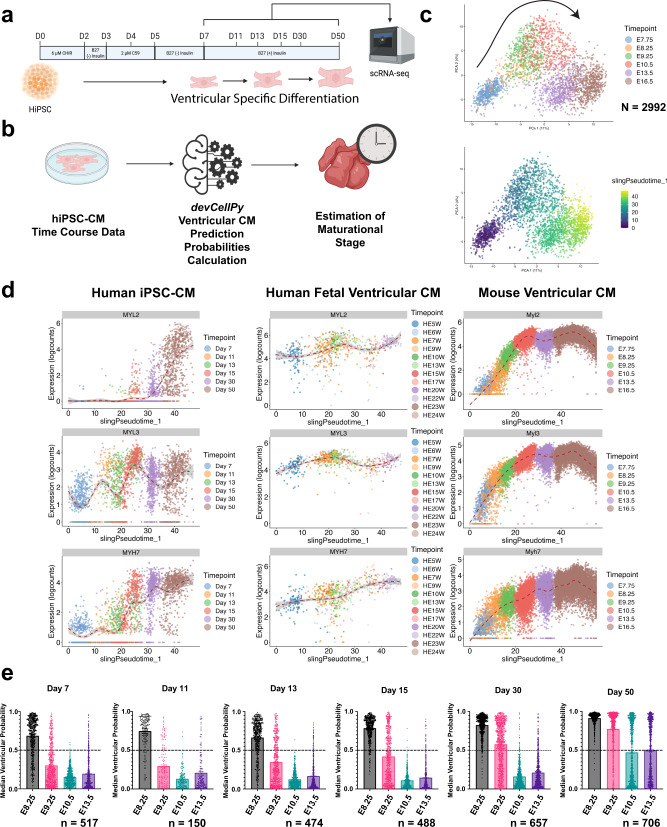
*devCellPy* murine prediction models identify developmental immaturity of ventricular predominant HiPSC-CMs. Human iPSC-derived cardiomyocytes (hiPSC-CMs) were differentiated and dissociated at multiple timepoints for scRNA-seq and evaluation of maturational state using *devCellPy*. **a** Overview of protocol used for scRNA-seq time course of hiPSC-CMs. **b** Schematic of workflow for prediction of hiPSC-CM subtypes using *devCellPy* model trained on murine cardiomyocyte data. **c** Plots of hiPSC-CM scRNA-seq time course data along the first two principal components. Top, plot illustrates the transition of cardiomyocytes from early day 7 followed by a progressive transition to day 50. Bottom, plot illustrates pseudotime demonstrating developmental progression. **d** Gene expression changes across pseudotime for three ventricular markers *MYL2, MYL3, and MYH7* across human iPSC, human fetal, and mouse ventricular cardiomyocytes. **e** Median ventricular cardiomyocyte prediction probabilities computed by *devCellPy* for murine models used for predicting the ventricular identity of hiPSC-CMs. Error bars represent 95% confidence intervals. Number of cells predicted per timepoint included Day 7 = 517, Day 11 = 150, Day 13 = 474, Day 15 = 488, Day 30 = 657, Day 50 = 706.

Having validated the accuracy of *devCellPy* in classifying embryonic mouse cardiac cells, we asked whether we could use our murine cardiac prediction algorithm to accurately predict the identity of human-induced pluripotent stem cell (hiPSC) derived cardiomyocytes (Fig. 7a, b). Importantly, we asked whether early embryonic mouse models were better predictors of cardiomyocyte subtypes in the hiPSC system given the known immaturity of in vitro-derived cardiomyocytes. We differentiated hiPSCs using a standard biphasic WNT modulation protocol and conducted a time coursed scRNA-seq profiling starting from day 7 and ending with day 50 of differentiation (Fig. 7a). We conducted an analysis of cardiomyocytes across six timepoints and plotted single cells across the first two principal components. As expected, cells progressed along^58–61^ the first principal component in a time-dependent manner (Fig. 7c). Using Day 7 as the starting point, the Slingshot package was used to calculate pseudotime, a continuous variable that measures developmental progression within a single cell dataset. Previously, it has been reported that small molecule biphasic WNT protocols predominantly give rise to ventricular-specific cardiomyocytes in the absence of posteriorizing retinoic acid signaling^58,61–63^. To confirm this, we plotted the expression of validated ventricular markers *Myl2*, *Myl3*, *Myh7* during hiPSC, human fetal^39^, and mouse embryonic development and confirmed the gradual increase in expression of all of these definitive ventricular markers (Fig. 7d).

Having established the ventricular-specific differentiation of our hiPSC-CMs, we next asked whether *devCellPy* models trained on murine data could accurately identify the ventricular identity of our hiPSC-CMs. We trained multiple *devCellPy* prediction models for identifying cardiomyocyte subtypes based on early murine embryonic timepoints between E7.75 and E13.5. The timepoint-based models were then used for computing the prediction confidence of *devCellPy* for assigning hiPSC-CMs a ventricular identity (Fig. 7e). The cross-species prediction was conducted by converting human genes in the hiPSC-CM dataset to homologous mouse genes to apply the *devCellPy* murine models. Interestingly, we found that across all days of hiPSC cardiac differentiation, the E8.25 mouse model provided the highest median prediction probability across all timepoints analyzed. As hiPSC differentiation progressed, the ventricular probabilities of the E9.25–E13.5 gradually increased consistent with the maturation of the hiPSC-CMs. Overall, however, only E9.25 reached above the 50% cutoff threshold used by *devCellPy* for making a confident prediction. We also tested *devCellPy* model trained on human fetal data from 5 to 7 weeks of gestation and observed that human fetal data was a poor predictor of early timepoint hiPSC-CM similar to the E10.5–E13.5 murine models (Supplementary Fig. 12). Together, these data suggest that hiPSC-CMs exhibit developmental immaturity and are more confidently predicted by E8.25 murine data suggesting the embryonic phenotype of hiPSC-CMs and close conservation of murine cardiomyocyte maturation gene expression programs.

### TBX5 lineage tracing confirms the *devCellPy*-generated cardiac algorithm’s prediction of predominantly left ventricular cardiomyocyte identity of hiPSC-CMs

**Fig. 8:**
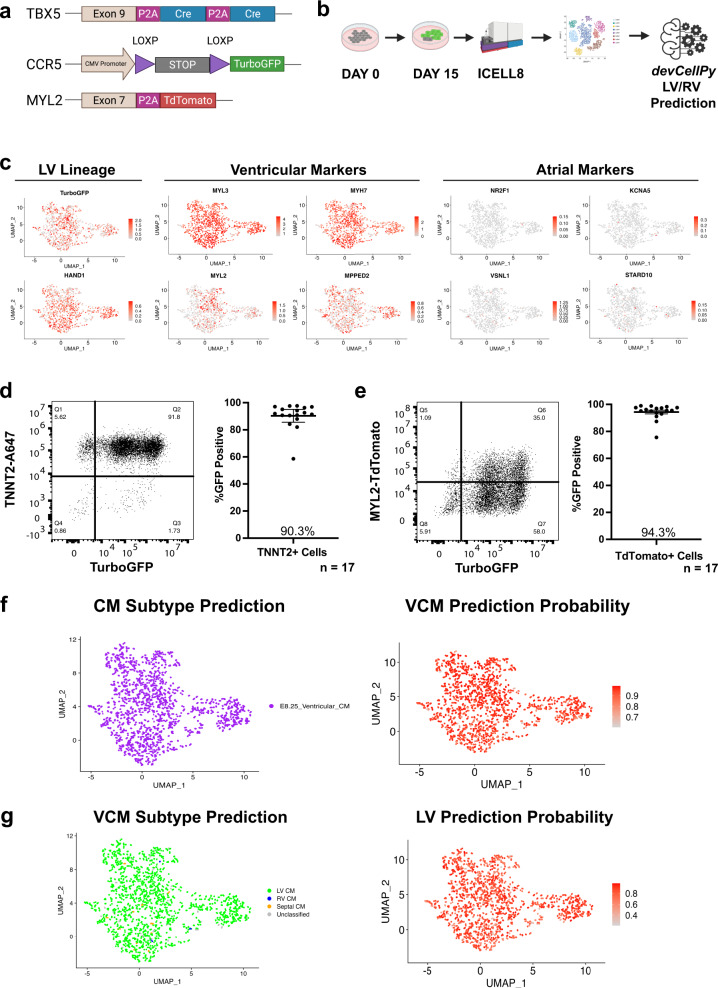
TBX5-Lineage tracing confirms *devCellPy* prediction of a predominantly left ventricular cardiomyocytes differentiation of hiPSC-CMs in vitro. A human iPSC line was genome-edited to introduce a *TBX5*-*Cre*/LoxP lineage tracing system. Genome-edited hiPSCs were differentiated and collected for scRNA-seq using the ICELL8 Smart-seq2 system. *devCellPy* models used for murine ventricular cardiomyocyte subtype predictions were applied for the prediction of the hiPSC-CMs. **a** Schematic of dual *TBX5-Cre*/*MYL2-TdTomato* lineage tracing system. **b** workflow for collection of day 15 ICELL8 data from genome-edited cell line. **c** Feature plots showing the expression of *TurboGFP*, LV marker *HAND1*, ventricular markers *(MYL3, MYL2, MYH7, MPPED2),* and atrial markers (*NR2F1, KCNA5, VSNL1, STARD10)*. **d** Left, Representative flow cytometry plot of cardiac troponin T (TNNT2) and TurboGFP expression at day 35 of cardiomyocytes containing *TBX5*-lineage tracing system. Right, Quantification of GFP-positive percentage among TNNT2 + cardiomyocytes among 17 independent biological replicate differentiations. Error bars represent standard error around the mean. **e** Left, Representative flow cytometry plot of MYL2-TdTomato and TurboGFP expression at day 35 of cardiomyocytes. Right, Quantification of GFP-positive percentage among TdTomato+ cardiomyocytes among 17 independent biological replicate differentiations. Error bars represent standard error around the mean. **f** Predictions of cardiomyocyte subtypes for day 15 hiPSC-CM using *devCellPy* E8.25 model and prediction probabilities. **g** Predictions of ventricular cardiomyocyte subtypes (left) of day 15 hiPSC-CM and prediction probabilities (right). Prediction probabilities indicate high overall confidence in LV predictions.

Since our hiPSC-CMs were shown to be almost entirely ventricular at late stages of development, we asked whether the *devCellPy*-generated cardiac algorithm could accurately predict their ventricular cardiomyocyte subtype identity (e.g. RV, LV, septal). To validate the *devCellPy* predictions, we used a dual *TBX5*-targeted Cre-LoxP/MYL2-TdTomato lineage tracing system in an hiPSC line that we previously developed to label LV cardiomyocytes in vitro (Fig. 8a)^64^.*TBX5* expression is well-known to be restricted to LV but not RV cardiomyocytes and their precursors in murine models^33^. We differentiated an hiPSC line containing the lineage tracing system to day 15 cardiomyocytes and harvested the cells for scRNA-seq study using the ICELL8 Smart-seq2 system (Fig. 8b). Surprisingly, we observed that a majority of cardiomyocytes expressed *TurboGFP* (i.e. as *TBX5* lineage descendant) and *HAND1* by day 15 indicating the FHF predominance of differentiation in this hiPSC line (Fig. 8c). Moreover, we observed the high expression of ventricular markers (*MYL3, MYH7, MYL2, MPPED2*) and almost no expression of atrial markers (*NR2F1, KCNA5, VSNL1, STARD10*) indicating the putative left ventricular identity of these hiPSC-CMs. Flow cytometry analysis of day 35 differentiated hiPSC cardiomyocytes containing the *TBX5* lineage tracing system revealed 90.3 ± 2.3% of TNNT2+ cells (Fig. 8d) and 94.3 ± 1.4% of ventricular cardiomyocytes marked by TdTomato expression were GFP+ (Fig. 8e). These results are consistent with day 15 scRNA-seq data showing a predominance of left ventricular gene marker expression.

Having observed a predominance of LV differentiation of hiPSCs, we next asked whether *devCellPy* could be used to predict the cardiomyocyte chamber identity of hiPSC-CMs from scRNA-seq data. We chose to conduct a cross-species prediction of ventricular chamber identity due to the lack of available early-stage human embryonic cardiac cell data that include cells earlier than 5 weeks of human development. Using the E8.25 prediction model, *devCellPy* classified 100% of all cardiomyocytes as ventricular subtypes with >90% prediction probabilities (Fig. 8f). Moreover, the majority of cells were predicted to be of LV identity with >80% prediction probabilities across most cells (Fig. 8g). Overall, these results provide an intriguing finding demonstrating that *devCellPy* can be utilized for cross-species cell type predictions of in vitro hiPSC-derived cells.

## Discussion

In this study, we developed *devCellPy*, a Python-based bioinformatics pipeline to automatically predict cell types across multiple timepoints of development and cascading tiers of annotation layers using an accurately curated reference dataset at high resolution. Hierarchical annotation structures are present across multiple organs, species, and model systems^1,5,22,65–68^. As illustrated by the cardiac developmental atlas presented here, cells exhibit multiple subclasses of identities including ones that are difficult to resolve without extensive sub-clustering or sub-setting of the data. Moreover, accurate manual annotation of datasets often requires expert knowledge of the literature on positively and negatively expressed markers, which may lead to poor reproducibility in dataset annotations in less experienced users. Previous studies have also shown that deeply annotated datasets that contain highly related cell types with multiple subclasses can negatively impact the performance of automated cell classifiers^69^. We approached this challenge by creating hierarchically organized prediction models that encode their positions within an annotation hierarchy in an object class we term a “LayerObject”. By breaking down the prediction of closely related cell types into distinct annotation layers, we aimed to achieve a higher resolution prediction outcome across highly granular subclasses of cells within the annotation hierarchy. Importantly, users can designate timepoints within the hierarchy allowing for the construction of timepoint-dependent predictions across multiple sublayers of annotation. As cells move through the prediction hierarchy, *devCellPy* will only proceed to the next level of classification for cells passing the probability threshold set by the user, thus allowing for high confidence annotations of cell types across each layer of classification. Moreover, LayerObjects within *devCellPy* are highly portable allowing users to share trained prediction models or to export individual LayerObjects for single-layer predictions. The portability of *devCellPy* allows expert-curated reference atlases to be widely available to the scientific community for conducting cell predictions across complex scenarios such as developmental-specific cell predictions.

In addition to its portability, *devCellPy* requires minimal processing of a dataset for training and prediction. Surprisingly, we found that *devCellPy* could identify biologically relevant marker genes across more than 35,000 input genes. The high prediction performance of the algorithm revealed that no additional preprocessing or feature selection was required other than the normalized counts expression matrix to train the algorithm and reference labels from an accurately curated reference dataset at high resolution. Importantly, *devCellPy* significantly reduces the burden on users to conduct extensive dimensionality reduction analyses and prior feature selection as required for other classification tools^12,15,16^ Interestingly, when we compared the performance of *devCellPy* to other previously published cell classification algorithms, we found that the automated hierarchical approach of *devCellPy* outperformed other methods trained on classifying all the cell types present within the cardiac atlas (Fig. 6). A major limitation of existing methods for predicting highly granular cell types is that the requirement of feature selection in the gene expression or principal component space may filter out genes necessary to predict cell subtypes. While feature selection has been shown to improve prediction accuracies^13,43^, the presence of highly granular cell subtypes can yield inaccurate classification and requires users to conduct laborious partitioning and reselection of significant features for cell prediction. *devCellPy* fully automates the training across multiple levels of cell identities thus automating the process of predicting cells across complex annotation hierarchies. Moreover, while other methods allow for the generation of cell prediction models for a unique set of labels, *devCellPy* allows for automated classification across timepoint-dependent annotations, thus providing a significant improvement for cell classification across developmental datasets. Importantly, *devCellPy* was able to accurately predict cell types from as low as 32 cells per category without the need for feature selection or calculation of correlated features prior to feeding the dataset to *devCellPy* which are otherwise implemented in algorithms such as CaSTLe or scmap.

To demonstrate the broad utility of *devCellPy* on cells from any species, we show its ability to accurately predict de novo datasets regardless of the single cell technology used for dataset generation. One example of this was seen with the Lescroart et al. dataset where Smart-seq data was collected on gastrulation stage embryos^50^. *devCellPy* was able to predict all cells for this dataset with a nearly 100% match to manual annotations despite being trained on data gathered from 10X Genomics 3’ droplet-based data. Furthermore, the *devCellPy-*generated cardiac algorithm successfully predicted RV cardiomyocytes marked by an *Isl1*-lineage tracing marker. The concordance of the algorithm’s predictions with a fluorescent lineage positive marker provides evidence for the overall accuracy of the algorithm at cell prediction of highly granular cell types such as ventricular cardiomyocyte subtypes.

Despite a more than 15-year history of hiPSC cardiac differentiation, it remains unknown whether hiPSCs generate left or right ventricular cardiomyocytes^58,60^. This question is important for the modeling of congenital heart diseases such as single ventricle defects that lead to devastating defects where one side of the heart is severely underdeveloped. Moreover, a major challenge in the field of hiPSC cardiac biology has been to generate distinct types of cardiomyocytes and to identify them in vitro^61,62,70^. We, therefore, applied *devCellPy* to determine whether the algorithm could identify the cardiomyocytes generated from in vitro differentiated hiPSCs by using the murine mouse models. Intriguingly, the *devCellPy*-generated algorithm identified the developmental immaturity of hiPSC-CMs similar to E8.25 between Day 7 and 30 of maturation, with Day 50 being accurately predicted by the E9.25 model. These data thus suggest that hiPSC-derived CMs exhibit an embryonic-like maturational state. Moreover, *devCellPy* models trained on 5–7 weeks of human fetal heart data showed poor prediction confidence for almost all early timepoints of hiPSC differentiation which highlights the need for an early embryonic reference similar to E8.25–E9.25 in the mouse (Supplementary Fig. 12).

Interestingly, *devCellPy* predicted the majority of the hiPSC-derived CMs as left ventricular in identity. To confirm this, we introduced a TBX5-driven Cre-LoxP lineage tracing system in an hiPSC line^64^, which corroborated the prediction of the machine learning algorithm, thus providing evidence of left ventricular predominant differentiation in an hiPSC cell line. The ability to conduct a cross-species prediction was particularly intriguing due to the lack of embryonic stage human embryonic tissue that could be used as a reference. As groups continue to generate increasingly complex in vitro differentiation systems such as organoids^67,71–73^, *devCellPy* should be able to serve as a useful tool to identify multiple classes of cell types and enable cross-species prediction of early embryonic cell types.

Together, *devCellPy* presents an exciting tool for scRNA-seq analysis by providing a fully automated pipeline for generating a cell type/subtype prediction algorithm that is well suited for hierarchically annotated datasets. Our work here shows that the algorithm generated by *devCellPy* is highly versatile and generalizable to any scRNA-seq dataset and is provided as a fully open-source Python package. With the growth of large-scale developmental cell atlases, *devCellPy* will provide a resource to assist in the identification of cell types across platforms and species, particularly in well-annotated reference datasets exhibiting complex multilayered annotation schemes.

## Methods

### Animal samples

All animal experiments described have been approved by the Administrative Panel on Laboratory Animal Care at Stanford University. Mouse strain used for all experiments was the CD1 wildtype background. Time pregnancies of CD1 were acquired from Jackson Laboratory (Sacramento, CA) and 8 embryonic day 10.5 mice of mixed sex were used for single-cell experiment in this manuscript.

### Cell lines

The human-induced pluripotent stem cell (hiPSC) line used in this study was provided by the Stanford Cardiovascular Institute Biobank (SCVI-111, Sendai virus reprogrammed peripheral blood mononuclear cells, healthy male with normal karyotype 46, XY). Genome edited SCVI-111 containing TBX5-Cre lineage tracing/MYL2-Tdtomato system were previously generated as described in ref. 64. Studies involved human iPSC approved under protocol #460 of the Stanford Stem Cell Research Oversight (SCRO) committee.

### Cell culture

HiPSCs were maintained in DMEM/F12 (Thermo Fisher Cat. 11330057) supplemented with Essential 8 (E8) and cultured on growth factor reduced Matrigel (Corning Cat. 354230) coated plates at a 1:350 dilution. Upon reaching 75–80% confluency, hiPSCs were passaged using 0.5 mM EDTA in PBS for 8 min at 37 °C. Passaging was conducted by trituration of the dissociated cell clustered in E8 media plus 10 µM ROCK inhibitor (Selleckchem Cat. S1049). Passaging was performed in 1:12 splitting ratios approximating 10,000 cells per cm^2^. 24 h after passaging, media was changed to E8 media without ROCK inhibitor.

HiPSC cardiomyocyte differentiation was performed using a previously described biphasic WNT stimulation and inhibition protocol as previously described^74^. Briefly, on day 0 of differentiation when hiPSCs reached 90–95% confluency, media was changed to RPMI 1640 (Thermo Fisher Cat. 11875119) differentiation media supplemented with B27 minus insulin (Thermo Fisher Cat. A1895601) containing 6 µM CHIR99021 (Selleckchem Cat. S1263). After 2 days of CHIR treatment, the media was changed to RPMI 1640 with B27 minus insulin (Thermo Fisher Cat. for 24 h. Between days 3 and 5, the media was changed to 2 µM C59 (Selleckchem Cat. S7037) in RPMI 1640 media supplemented with B27 minus insulin (Thermo Fisher Cat. A1895601). On day 5 of differentiation, media was changed to B27 minus insulin in RPMI 1640 for 48 h on day 7, media was changed to B27 plus insulin in RPMI 1640. On day 9, cardiomyocytes were enriched by glucose deprivation using RPMI 1640 minus glucose supplemented with B27 plus insulin (Thermo Fisher Cat. 17504044). By day 11, >80% beating cardiomyocytes could be observed.

Cardiomyocytes were replated using TrypLE Select Enzyme 10X (ThermoFisher Cat. A1217701) at 37 °C for 10–15 min. Cells were dissociated using gentle trituration and transferred to 15 mL conical tubes containing a cardiomyocyte replating media (10% knockout serum in RPMI 1640 supplemented with B27 plus insulin and 10 µM ROCK inhibitor). 24 h after plating cardiomyocytes, the media was changed to RPMI 1640 supplemented with B27 plus insulin, and cardiomyocytes were subsequently maintained in this media. Biological replicates were defined as independent differentiations conducted during experiments.

### Overview of devCellPy

*devCellPy* is a multilayered machine learning pipeline powered by the XGBoost Python library for cell classification. A fully annotated reference dataset containing individual cell annotations across all layers of annotation is required to train the program. Under train mode, *devCellPy* intakes a log-normalized counts matrix, cell annotations, and a file containing the annotation hierarchy. Hierarchies loaded into the software can be of any complexity and can include layers such as the time where *devCellPy* will train to recognize cell types that are only present during specific timepoints during a biological process. We created an object class that we term the LayerObject which will be generated for each layer of annotation within the hierarchy. At the beginning of training, XGBoost training parameters are fine-tuned at the topmost layer of the hierarchy using a randomized search with 50 trials. Learning rate, maximum depth, observation ratio per tree, and feature ratio per tree are adjusted to maximize accuracy and minimize overfitting. Final parameters are selected according to the minimum mean absolute error.

After the selection of the final model parameters, *devCellPy* will begin training XGBoost models for each layer of classification. Each layer is trained independently on subsets of the scRNA-seq data, which can be customized to identify different networks of labeling. The XGBoost models are stored within the LayerObjects of each respective layer. The LayerObject will also automatically encode the layer’s position within the hierarchy to allow the algorithm to automatically predict cell classes according to the desired annotation hierarchy. The final trained model will provide an output containing multiple LayerObjects that are exportable for reusability of the prediction model. LayerObjects can also stand alone allowing users to conduct individual layer cell annotations.

For cell prediction, *devCellPy* takes a log-normalized gene expression matrix that is then used to conduct cell predictions across all layers of annotation. Cells will only be classified according to the annotation hierarchy used for the training of the algorithm. At each layer of classification, a probability value indicating the confidence of a cell’s annotation is provided for each prediction made. We implemented a rejection option into the layered model with a default probability threshold of 0.5, and cells whose label with maximum probability falls under 0.5 are categorized as unclassified. In the final version of *devCellPy* this threshold value is adjustable by the user who may choose more stringent cutoffs for cell classification.

*devCellPy* also allows users to identify the top positive and negative gene predictors used by the algorithm to predict cell types. The contribution of each gene toward the prediction of each class was calculated based on the classic game-theoretic Shapley values using the SHapley Additive exPlanations (SHAP) package^23^, and the top genes with the highest Shapley value sum for each label were reported. SHAP importance plots for each cell type indicate top genes that either positively or negatively shifted XGBoost’s prediction toward a particular cell type. Using the feature ranking mode within *devCellPy*, top positive and negative genes used for the predictions of cell types across all layers will be generated thus allowing for the automated determination of gene markers used for cell type assignments by the algorithm.

For more information and a full tutorial of *devCellPy’s* functionality, we provide documentation at: https://github.com/devCellPy-Team/DevCellPy.

### Construction of cardiac developmental cell atlas

To generate a large-scale cardiac developmental cell atlas raw FASTQ files were downloaded from the GEO repository—deSoysa et al. 2021, Hill et al. 2021, and Goodyer et al. 2021—and aligned using CellRanger-6.0.0. Accession numbers can be found in Supplementary Table 1. We conducted a single-cell RNA-seq analysis using Seurat v4.0.3. Dataset quality control was conducted by excluding cells where the number of genes, number of mRNA counts, percent mitochondrial gene expression, or percent ribosomal gene expression exceeded the median of these metrics by plus or minus three times the median absolute deviation. An ambient RNA correction was conducted on all datasets using the SoupX package^75^. Following quality control, each dataset was normalized separately where each gene’s count was divided by the total number of gene counts per cell and scaled by a factor of 10,000. Normalized counts were then further processed by taking the natural log of each normalized gene count.

For each individual dataset, we proceeded to find the top 2000 highly variable genes using the FindVariableFeatures in Seurat. We performed a linear regression (using ScaleData function in Seurat) on all highly variable genes to eliminate technical variability due to the number of genes detected, sequencing depth, percent mitochondrial content, percent ribosomal content, and cell cycle phase. After scaling highly variable genes, these features were used as input for principal component analysis. Following principal component calculation, we used significant principal components to proceed with dimensionality reduction by uniform manifold approximation and projection (UMAP) and unsupervised clustering^76^.

During our first pass annotation, we selected all mesoderm-derived cell types relevant for cardiac development and excluded endodermal and ectodermal-derived cell types for subsequent analysis. After the selection of mesoderm derivatives, we re-clustered all cell types and selected a clustering resolution that allowed for the clear annotation of distinct cell types. We divided our annotation strategy into three layers of annotation. The first layer consisted of identifying major cell type categories (i.e. cardiomyocytes, endothelial cells, etc.). The second layer consisted of subsetting cardiomyocytes only and re-clustering all cells to identify major cardiomyocyte subpopulations. Using markers established in the literature we proceeded to annotate unsupervised clusters based on their cardiomyocyte subtype identity. Lastly, for the third layer of annotation we subsetted ventricular cardiomyocytes and re-clustered the cells to annotate ventricular cardiomyocyte subtypes. Across all three layers, we conducted differential expression analysis using pairwise comparisons across all cell classes using the Wilcoxon rank-sum test for single-cell gene expression. Cell markers identified during differential expression analysis were cross-referenced with established literature including original papers from which datasets were obtained to confirm cell identity.

To generate a unified differentiation trajectory starting from pluripotent epiblast cells, we downloaded the count's matrix from the ref. 2 gastrulation cell atlas and subsetted cells that were annotated as epiblast, primitive streak, nascent mesoderm, and mixed mesoderm. After normalization, we proceeded to integrate all datasets together using the mutual nearest neighbor (MNN) algorithm^28^. Using MNN-corrected principal components, we conducted dimensionality reduction and generated a UMAP plot demonstrating a continuous differentiation trajectory from early epiblast cells to distinct cardiac developmental cell types. We followed the same procedure to obtain a differentiation trajectory of FHF and aSHF progenitor differentiation to LV and RV cardiomyocytes, respectively. For visualization of the FHF/aSHF differentiation trajectory, we plotted three-dimensional UMAPs to visualize the bifurcation of the progenitor population from mesodermal progenitors.

### Mouse embryonic heart scRNA-seq workflow

E10.5 murine hearts were dissected from the thoracic cavity of a single litter. Whole hearts were then digested into single cells with 1 mL of 0.25% trypsin–EDTA at 37 °C for 10 min with continuous gentle trituration using a 1000 μL pipette. An equal volume of a collagenase mixture was subsequently added (10 mg/mL of collagenase A + 10 mg/mL of collagenase B dissolved in HBSS minus calcium with 40% FBS). Cells were again manually dissociated collagenase mixture for 20 min with gentle trituration using 1000 μL pipette. After dissociation, a large excess volume of HBSS minus calcium with 40% FBS was added to dilute and quench the enzyme reaction. Cells were subsequently centrifuged for 5 min at 200×*g* at 4 °C. Cells were resuspended in cold PBS + 0.04% BSA and washed twice in similar fashion prior to cell counting using a Countess cell counter. 10X Genomics Chromium Next GEM Single Cell 3’ v3.1 (Rev C) protocol was followed for single-cell capture and subsequent cDNA library generation. Input cell concentration was adjusted to achieve an input cell number of 10,000 cells according to the manufacturer’s protocol. Sequencing was conducted on an Illumina NovaSeq 6000 at 20,000 read pairs per cell for the gene expression library and 5000 read pairs for the feature barcode library.

### Human iPSC time course scRNA-seq workflow

We conducted a time course scRNA-seq experiment using the parental line of the hiPSC line containing the TBX5-Cre lineage tracing reporter system. Human-induced pluripotent stem cells were maintained and differentiated to cardiomyocytes using the protocol described above in the “Cell Culture” section. To conduct a time course scRNA-seq experiment we froze hiPSC-CMs on day 7 of differentiation and conducted timed thaws to synchronize the collection of cells on days 7, 11, 13, 15, 30, and 50 of differentiation. This was done to minimize experimental variability and to ensure that we followed the differentiation of a single differentiation batch through development. Cells were dissociated using TRYPLE Select 10X for 10–15 min with gentle trituration using a 1000 µL pipette tip every 3 min. Enzymatic digestion with replating media (10% KOSR in RPMI 1640 supplemented with B27 plus insulin and 10 µM ROCK inhibitor). Cells were filtered through a 100 µm filter and were counted to obtain 500,000 cells per timepoint. Cells for each individual timepoint were incubated with a distinct 10X Genomics CellPlex oligo following the manufacturer's protocol (CG000391 Rev A). CellPlex allows for multiplexing of samples for scRNA-seq experiments. Following CellPlex incubations and washes, we pooled all timepoints together at equal ratios and counted cells on a Countess (Invitrogen) automated cell counter. We then followed the 10X Genomics Next GEM Single Cell 3’ Reagent Kit v3.1 with Feature Barcode technology for Cell Multiplexing protocol (CG000388 Rev B) to generate a cDNA library. An estimated 50,000 cells were loaded onto the cell capture chip. The final products from the manufacturer's protocols were two sequencing libraries: (1) Gene expression library and (2) Feature barcode library for sample demultiplexing. Sequencing was conducted on an Illumina NovaSeq 6000 at 20,000 read pairs per cell for the gene expression library and 5000 read pairs for the feature barcode library. GEO Accession numbers for data are found in Supplementary Table 2.

### Bioinformatic analysis of human iPSC time course scRNA-seq data

Raw FASTQ files for the gene expression and CellPlex libraries were input into the CellRanger-6.0.0 “multi” function to align and demultiplex samples. CellRanger conducts demultiplexing of samples by identifying single oligo labeled cells for each sample and discarding cells that presented with multiple oligo labels which represent either doublets or background labeled cells. We conducted a subsequent analysis using the R package Seurat v4.0.5. After demultiplexing, each timepoint was individually pre-processed by conducting quality control by excluding cells where the number of genes, number of mRNA counts, percent mitochondrial gene expression, or percent ribosomal gene expression exceeded the median of these metrics by plus or minus three times the median absolute deviation. Counts for each cell were normalized by dividing each gene’s mRNA counts by the total number of counts expressed per cell. The normalized gene counts were then scaled by a factor of 10,000 and a natural log was calculated for each gene. We used a panel of cell cycle genes associated with the S, G2, and M phases to calculate a cell cycle score and assign the cell cycle state of each cell. 2000 Highly variable genes were calculated and technical variables (total gene counts, number of features detected, percent mitochondrial genes, percent ribosomal genes, and cell cycle status) were regressed out using linear regression. Principal component analysis was conducted followed by non-linear dimensionality reduction using uniform manifold approximation and projection (UMAP). Unsupervised clustering was conducted across all timepoints, and cardiomyocyte clusters were subsetted from each timepoint identified by the expression of canonical markers *TNNT2, ACTA2*, and *TNNI1*.

After identifying cardiomyocytes at each timepoint of differentiation, we subsetted all cardiomyocytes in the G1 phase of the cell cycle to eliminate the influence of cell cycle genes on downstream trajectory analysis of differentiation. All timepoints were merged into a single Seurat object and the object was converted into a SingleCellExperiment object for downstream trajectory analysis. Highly variable genes were calculated using the scran v1.18.5 package by using the modelGeneVar function and setting timepoint as a blocking variable to reduce the batch effect of individual timepoints^77,78^. Using the getTopHVGs function we then selected the top 2000 highly significant variable genes. We then removed cell cycle and ribosomal genes from this list to further reduce the effects of technical noise during the trajectory calculation. We conducted principal component analysis and used the top 2 principal components to plot cells and calculate pseudotime using the Slingshot v2.0.0 package. We calculated diffusion pseudotime and obtained a clear differentiation trajectory starting from day 7 of differentiation and progressing to day 50. Using the plotExpression function we were then able to plot gene expression of multiple genes across diffusion pseudotime and fit a generalized additive model curve for each gene’s expression.

### Human iPSC day 15 ICELL8 scRNA-seq workflow

Human iPSCs containing the TBX5 lineage tracing reporter system were differentiated to day 15 cardiomyocytes that were prepared for scRNA-seq using the same protocol as that used for the time course study. Cells were counted and resuspended in PBS + 0.04% BSA. We conducted single-cell capture and cDNA library generation using the Smartseq2 Takara ICELL8 single cell system (Takara Cat. No. 640000). Manufacturer's protocol was followed for single cell dispensing on the ICELL8 nanowell chip and automated microscopy was conducted to identify single cells for downstream processing. Each nanowell received individual index sequencing barcodes for demultiplexing cells during downstream sequencing. Sequencing per manufacturer instructions on an Illumina NovaSeq 6000 sequencer. GEO Accession numbers for data are found in Supplementary Table 2.

### Bioinformatic analysis of human iPSC-CM day 15 ICELL8 scRNA-seq

FASTQ files from human iPSC-CM day 15 ICELL8 experiments were aligned to a custom human reference genome containing TurboGFP sequence. Alignment was conducted using STAR-v2.7.9a using the following parameters: -soloType SmartSeq –soloStrand Unstranded –soloUMIdedup NoDedup^79^. The output from STAR was a gene expression matrix that was directly input into Seurat v4.0.3. We conducted data quality control, variable feature selection, and principal component analysis exactly as the process conducted for the hiPSC time course data. UMAP plots were calculated, and gene expression was analyzed using the FeaturePlot function in Seurat.

### Flow cytometry

A Beckman Coulter CytoFLEX flow cytometer was used for high throughput analysis of TNNT2, TurboGFP, and MYL2-TdTomato expression of hiPSC-CM derived from genome edited hiPSC containing the TBX5-lineage tracing reporter system. On days 3 and 35 of collection, cells were dissociated to single cells in 10X TrypLE Select (Thermo Fisher) for 5 min at 37 °C. Cells were subsequently pelleted by centrifugation at 200×*g* for 5 min. Cell pellets were resuspended in 4% PFA for 10 min and were rinsed with a 5% fetal bovine serum solution in 1× PBS. Cells were permeabilized in a 0.5% Saponin solution containing 5% FBS in 1× PBS (hereafter referred to as saponin solution). After permeabilization cells were incubated for 45 min in a monoclonal mouse anti-Troponin primary antibody (Thermo Fisher Cat. MA5-12960 Clone 13-11) at a 1:100 dilution in 0.5% saponin solution. Cells were rinsed twice in saponin solution and then incubated in secondary antibody AlexaFluor 647 goat anti-mouse (Thermo Fisher Cat. A-21235) at a 1:1000 dilution in 0.5% saponin solution. Cells were subsequently rinsed in 1× PBS twice and analyzed using CytoFLEX flow cytometer. Flow cytometry data was analyzed with FlowJo analysis software version 10.8.0. 23 independent biological replicates were analyzed for analysis of GFP expression and MYL2-TdTomato expression in hiPSC-derived cardiomyocytes as reported in Fig. 7 and Supplementary Fig. 8. Gating scheme was set relative to day 3 of differentiation which served as the control for analyzing fluorescent cell populations.

### Testing of devCellPy and other methods on Tabula muris dataset

Normalized expression data was obtained from the *Tabula muris* dataset and top 17 cell types containing >1000 cells were used for all analyses. The dataset was partitioned into 90% for cross-validation and 10% for a hold-out dataset that was used for testing across all machine learning methods. 10-fold cross-validation was conducted by randomly reshuffling the data used for training and testing to obtain a statistical estimate of each algorithm’s overall accuracy. Code for running each machine learning method was found in the following respective links: CaSTLe (https://github.com/yuvallb/CaSTLe), SingleCellNet (https://github.com/yuvallb/CaSTLe), Seurat (https://satijalab.org/seurat/articles/integration_mapping.html), scmap (https://github.com/hemberg-lab/scmap), scPred (https://github.com/powellgenomicslab/scPred). For the calculation of statistical significance of differences between methods evaluated, one-way Brown–Forsythe and Welch ANOVA tests were conducted.

### Determination of minimum cell number for *devCellPy* accuracy

Normalized expression data was obtained from the *Tabula muris* dataset. Cell types containing >1000 cells were selected for further testing. Dataset was partitioned into a 10% hold out dataset and 90% dataset that was used to randomly construct 5 partitions containing 16, 32, 64, 128, 256, 512, and 948 cells per cell type. *devCellPy* models were trained on each of these partitions for the number of cells per category and tested on the 10% hold-out dataset. Prediction accuracies were then calculated for all 5 models trained for each of the number cells per cell type. Confusion matrices were calculated on a representative partition to determine the overall accuracy per cell types.

### Testing of *devCellPy* on cardiac developmental atlas

*devCellPy* was used for classifying cells in the cardiac atlas consisting of the following structure: the first layer of classification organized cells based on their cell type, the second layer classifies cardiomyocyte cells, and the third layer classifies ventricular cardiomyocytes based on their location according to their developmental stage. To test the performance of the algorithm on each layer of annotation, each layer was trained independently on subsets of the scRNA-Seq atlas. For each layer tested, the data was divided into a 90% partition for cross-validation and a 10% hold-out dataset. Subsequently, the 90% cross-validation partition underwent further partitioning into a 90% segment used for training and a 10% segment used for calculation of performance metrics. 10-fold cross-validation was conducted by randomly reshuffling the data used for training and testing to obtain a statistical estimate of the model’s error for its overall accuracy, precision, recall, and F1 scores. After 10-fold cross-validation, we fed the 10% held-out partition from the initial subdivision and calculated the confusion matrices to determine the prediction accuracy of the algorithm across all cell classes.

### RNAScope in situ hybridization

Whole hearts were collected from embryonic day 10.5 and 16.5 CD1 mice and washed in PBS prior to overnight fixation in 4% paraformaldehyde (Fisher, 50-980-487) in PBS at 4 °C. Hearts were then washed in PBS at room temperature for 15 min three times prior to cryopreservation in 30% sucrose in PBS overnight at 4 °C. The tissue was then embedded in Tissue-Plus OCT (Fisher, 23-730-571) and was cut as cryosections of 10 µm thickness and stored at −80 °C until use. RNAscope© Multiplex Fluorescent v2 (Cat. #323100) was used per the manufacturer's suggested protocol for fixed frozen tissue sections. The following murine probes were used: Mm-Tbx5-C2 (Cat. #519581-C2) and Mm-Ppp1r17-C3 (Cat. #497351-C3). All images were taken with the Zeiss AxioImager widefield fluorescence microscope at the Neuroscience Microscopy Services facility at Stanford University. A minimum of three biological (different hearts) and three technical (different sections/heart) were used for each in situ hybridization.

### Validation of *devCellPy* on de novo datasets

For additional validation of the final *devCellPy* model, independent murine cardiac cell datasets were downloaded from refs. 80, 56, 57. Following the same procedure as that used for the cardiac developmental cell atlas, we conducted quality control, normalization, and a multilayered manual annotation of cell types. Moreover, we collected E10.5 mouse hearts and conducted scRNA-seq on these hearts to obtain additional data for testing *devCellPy* on never-before-seen data. We followed the same analysis procedure as the cardiac developmental cell atlas for manual annotation of distinct cell types. Normalized gene expression matrices were exported for each of these datasets and were input into *devCellPy* to conduct a full prediction of all cell types in the cardiac atlas. Confusion matrices were calculated to compare the manual labels versus the *devCellPy* predictions for all cells that were successfully classified. To evaluate *devCellPy* performance relative to other machine learning methods, we trained *SingleCellNet, Seurat*, and *scPred* methods on the full cardiac atlas labels and applied each method to the de novo datasets. Similar to *devCellPy*, we then calculated confusion matrices to compare the predicted labels to the manual annotations of distinct cell types.

To determine whether *devCellPy* could accurately predict *Isl1*-Cre lineage traced right ventricular cells, we calculated the number of ventricular cells in the Li et al. 2019 E10.5 dataset that were EGFP-positive cells by annotating cells that exhibited an expression level greater than 0.1. We then calculated the number of EGFP + cells that were classified as LV, RV, and Septal in both the manual and *devCellPy* annotation methods to determine the overall concordance between the classification methods.

### Analysis ventricular gene expression during ventricular differentiation

To conduct an analysis of atrial and ventricular marker expression during ventricular differentiation, we subsetted ventricular cardiomyocytes from both the human fetal and murine developmental cell atlas. We plotted each dataset along the first two principal components and calculated pseudotime using the Slingshot package. This same procedure was used for the hiPSC-CM time course. We then plotted the expression and fit generalized additive model curves for *MYL2, MYL3*, and *MYH7* to compare expression kinetics across all three datasets.

### Prediction of chamber identity of human iPSC cardiomyocytes

To predict the chamber identity of human iPSC cardiomyocytes using *devCellPy*, we exported the normalized expression matrix of the day 15 hiPSC-CM ICELL8 data. Human genes were converted to homologous mouse genes using *BioMart* package in R and we ran predictions of cardiomyocyte subtype identity using the E8.25 murine timepoint models. We then quantified the identities predicted using all three models for the two timepoints input into the prediction algorithm.

### List of R packages used in study

R (v4.1.1), Seurat (v4.0.5), SeuratWrappers (v4.0.2), SeuratObject (v4.0.2), SoupX (v1.5.2), celda (v1.8.2), ggplot2 (v3.3.5), ggpubr (v0.4.0), dplyr (v1.0.7), R.utils (v2.11.0), biomaRt (v2.48.3), svglite (v2.0.0), scran (v1.20.1), SingleCellExperiment (v1.14.1), scrattch.io (v0.1.0), and patchwork (v1.1.1).

### Reporting summary

Further information on research design is available in the Nature Research Reporting Summary linked to this article.

## Supplementary information

Supplementary Information

Reporting Summary

Description of Additional Supplementary Files

Supplementary Data 1

Supplementary Data 2

## Data Availability

The scRNA-seq data generated in this study have been deposited in the GEO Repository under accession code GSE184943. The publicly available datasets used in this study are available in the GEO Repository and ArrayExpress databases under accession codes GSE165300, GSE126128, GSE131181, GSE132658, GSE122403, GSE100471, E-MTAB-6967 (ArrayExpress). The cross-validation, flow cytometry, and minimum cell number determination data generated in this study are provided in the Source Data file. Source data are provided with this paper.

## Source data

Source Data

## Code availability

The code used for the generation of *devCellPy* is available at https://github.com/devCellPy-Team/devCellPy. *devCellPy* is available for download from the PyPi respository using the command “pip install devCellPy”. We provide full documentation of the code written for the development *devCellPy* in the *devCellPy* Github. The pipeline used for the construction of the cardiac scRNA-seq atlas is described in the “Methods” section and custom scripts are freely available upon request by contacting the corresponding author.
