# Supplementary material for: devCellPy is a machine learning-enabled pipeline for automated annotation of complex multilayered single-cell transcriptomic data
Source: Nat Commun. 2022 Sep 7;13:5271. doi: 10.1038/s41467-022-33045-x (PMC9452519; doi:10.1038/s41467-022-33045-x)
Supplement: Supplementary file 1 — Supplementary Information [file 41467_2022_33045_MOESM1_ESM.pdf]

SUPPLEMENTARY INFORMATION

devCellPy A machine learning-enabled pipeline for automated annotation of complex multilayered single-cell transcriptomic data

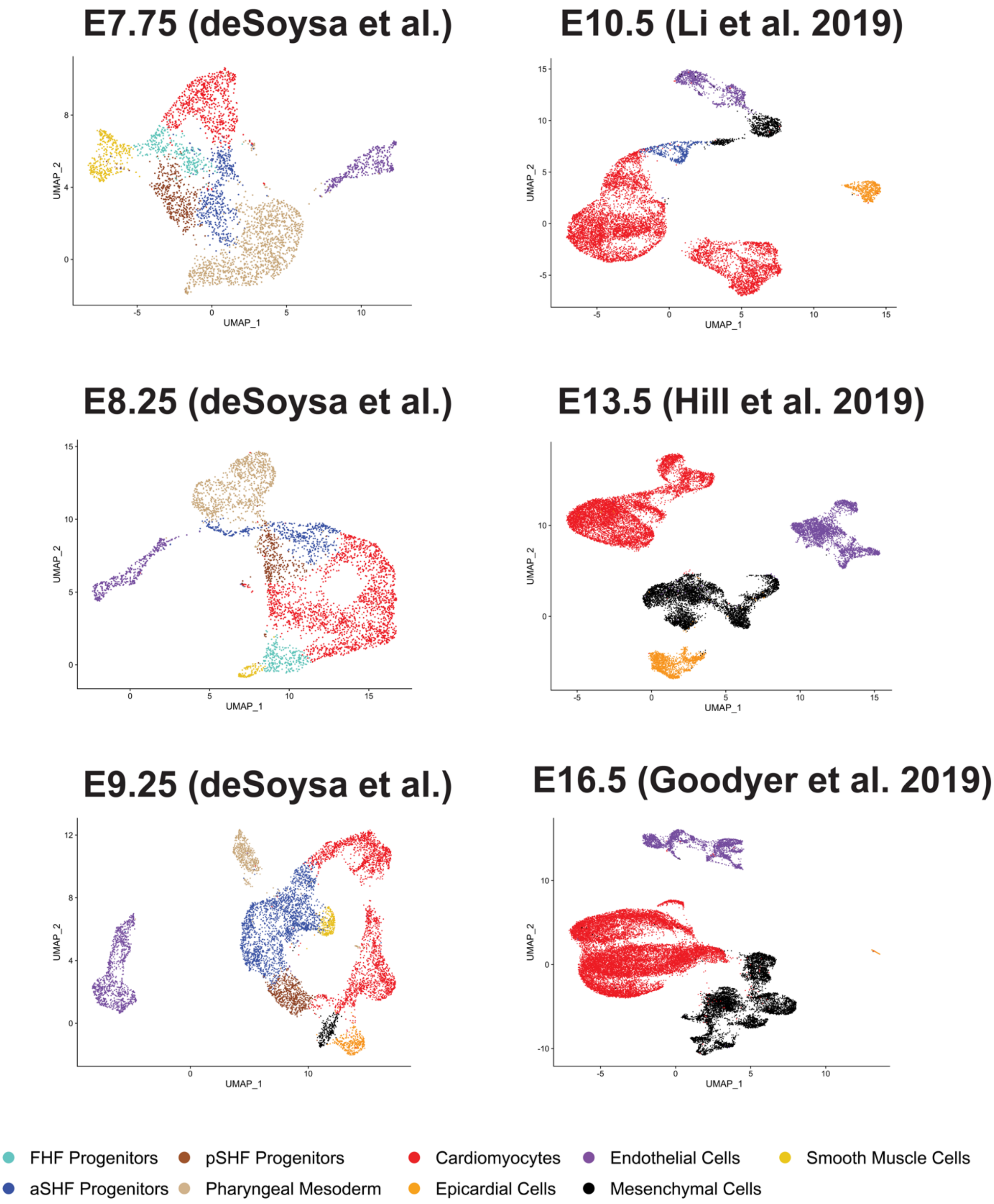

Supplementary Figure 1. Annotations of Unsupervised Clusters For Individual Datasets Included In Cardiac Developmental Atlas. Each individual timepoint for all downloaded datasets were analyzed independently and underwent unsupervised clustering. Using established gene markers and differential gene expression analysis, clusters were annotated as shown.

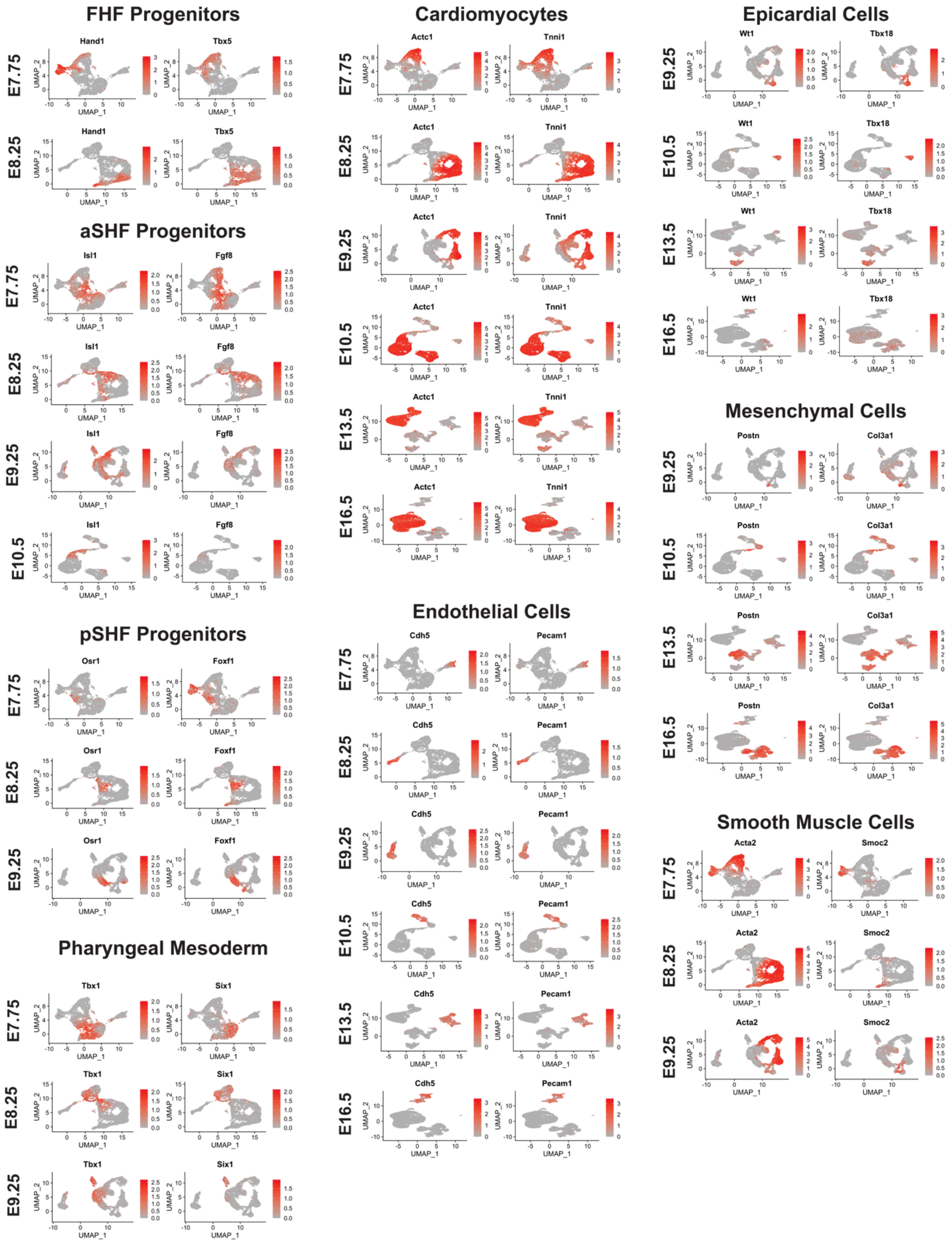

Supplementary Figure 2. Feature Plots Showing Top Gene Markers Used in Annotation of Cardiac Developmental Atlas. Feature plots showing the expression of well-established gene markers for multiple cell types included in the cardiac developmental atlas. Each major cell type class is divided into each individual timepoint showing the expression of distinct markers through time.

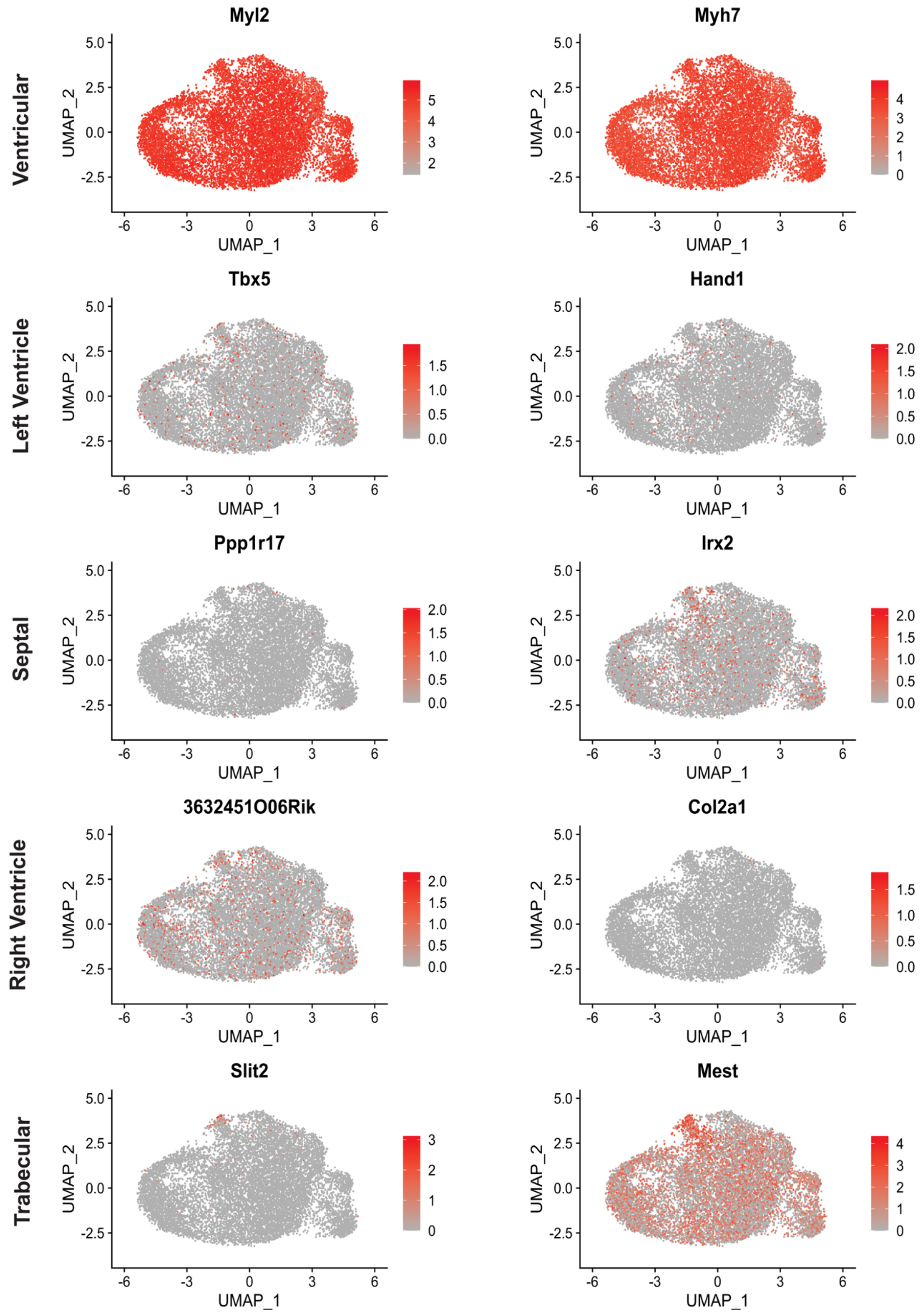

Supplementary Figure 3. Feature Plots Indicating Lack of Discernable Ventricular Cardiomyocyte Subtypes at E16.5. E16.5 ventricular cardiomyocytes were subclustered to determine whether LV, RV, Septal, Compact, or Trabecular cardiomyocyte subtypes could be identified. Two markers per ventricular

cardiomyocyte subtype are shown for each subclass of ventricular myocyte. Note the lack of clear expression in specific clusters of cells for all markers assayed.

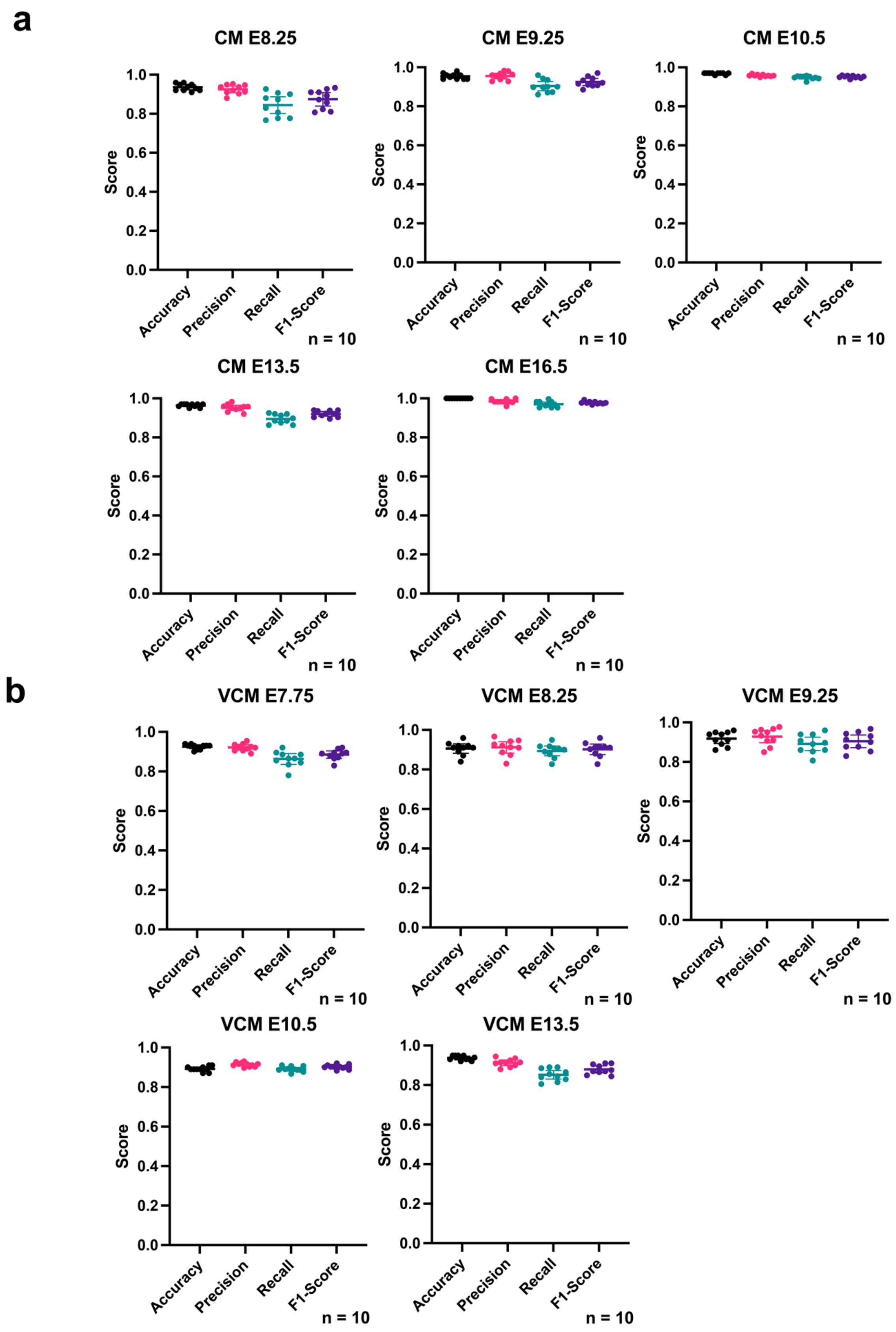

Supplementary Figure 4. Cross Validation Results for *devCellPy* Trained On Cardiac Atlas Layers 2 and 3. 10-fold cross validation results is presented for cardiomyocyte subtype layers. **A)** Cardiomyocyte subtype cross validation results presented by timepoints between E8.25-E16.5. **B)** Ventricular cardiomyocyte

subtype cross validation presented by timepoints between E7.75-E13.5. N = 10, for independent folds used for evaluation of all performance metrics across all timepoints. Error bars represent 95% confidence intervals.

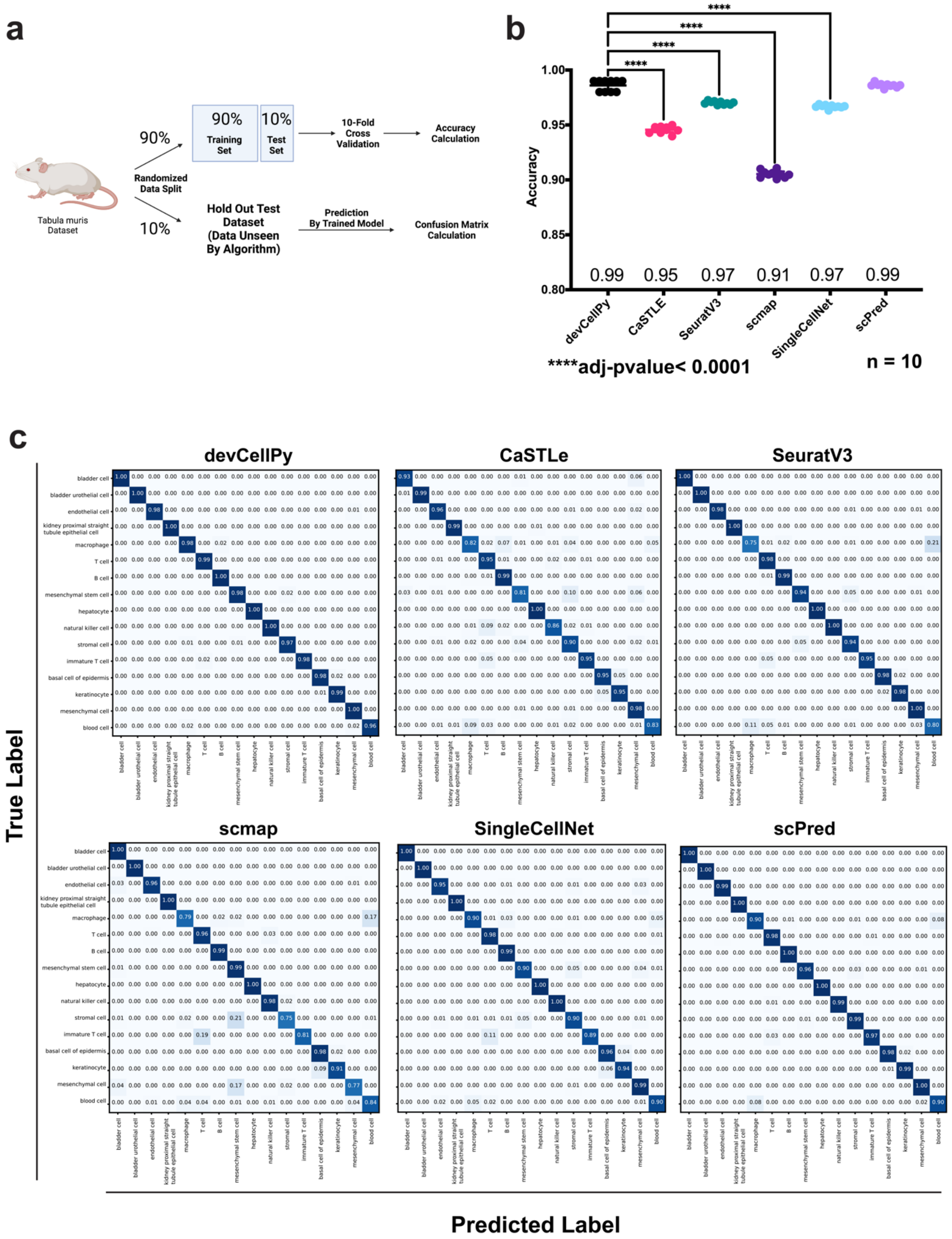

Supplementary Figure 5. Comparison of *devCellPy* to Previously Published Cell Prediction Algorithms. *devCellPy* performance accuracy was compared to other published machine learning methods using a subset of the *Tabula muris* dataset. **A)** Overview of cross validation and hold out dataset testing used for accuracy and confusion matrix calculations. **B)** 10-fold cross validation results for *devCellPy,* CaSTLe, SeuratV3, scmap, SingleCellNet, and scPred. N = 10 for independent folds tested for validation. Error bars represent 95% confidence

intervals. One-way Brown-Forsythe and Welch ANOVA tests with correction for multiple comparisons was conducted. ****Adjusted pvalue <0.0001.**C)** Confusion matrices for all algorithms evaluated using the same hold out dataset.

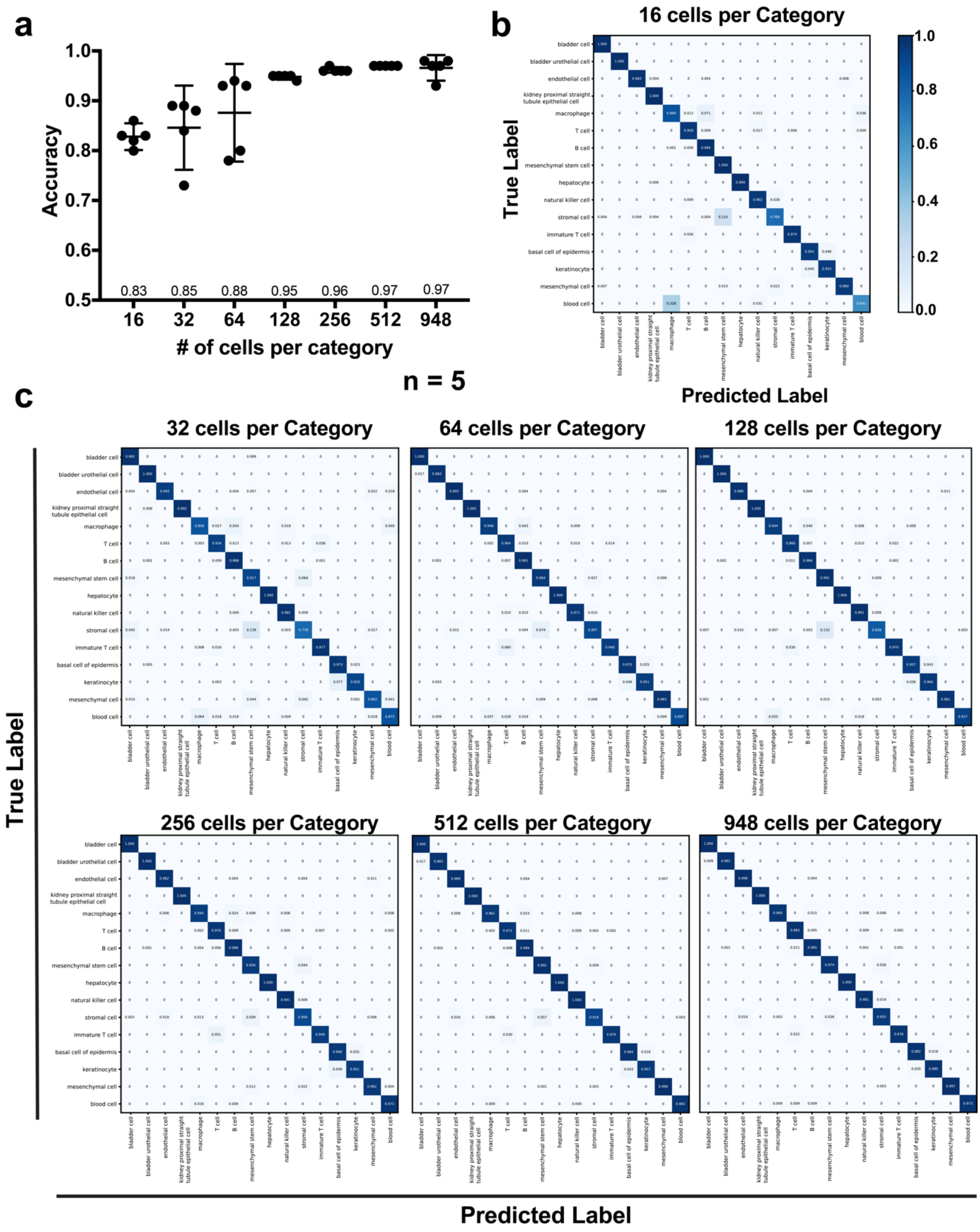

Supplementary Figure 6. Determination of Minimum Required Cell Number For Accurate *devCellPy* Predictions. Determination of the minimum number of cell types required to make accurate predictions using *devCellPy.* Error bars represent 95% confidence intervals. **A)** *devCellPy* was trained 5 independent times on with a minimum of 16, 32, 64, 128, 256, 512, and 948 cells per cell type and tested on a held out dataset. Mean cell accuracies are plotted. **B,C)** Confusion matrices for *devCellPy* models trained on varying number of cells per cell types of the *Tabula muris* dataset.

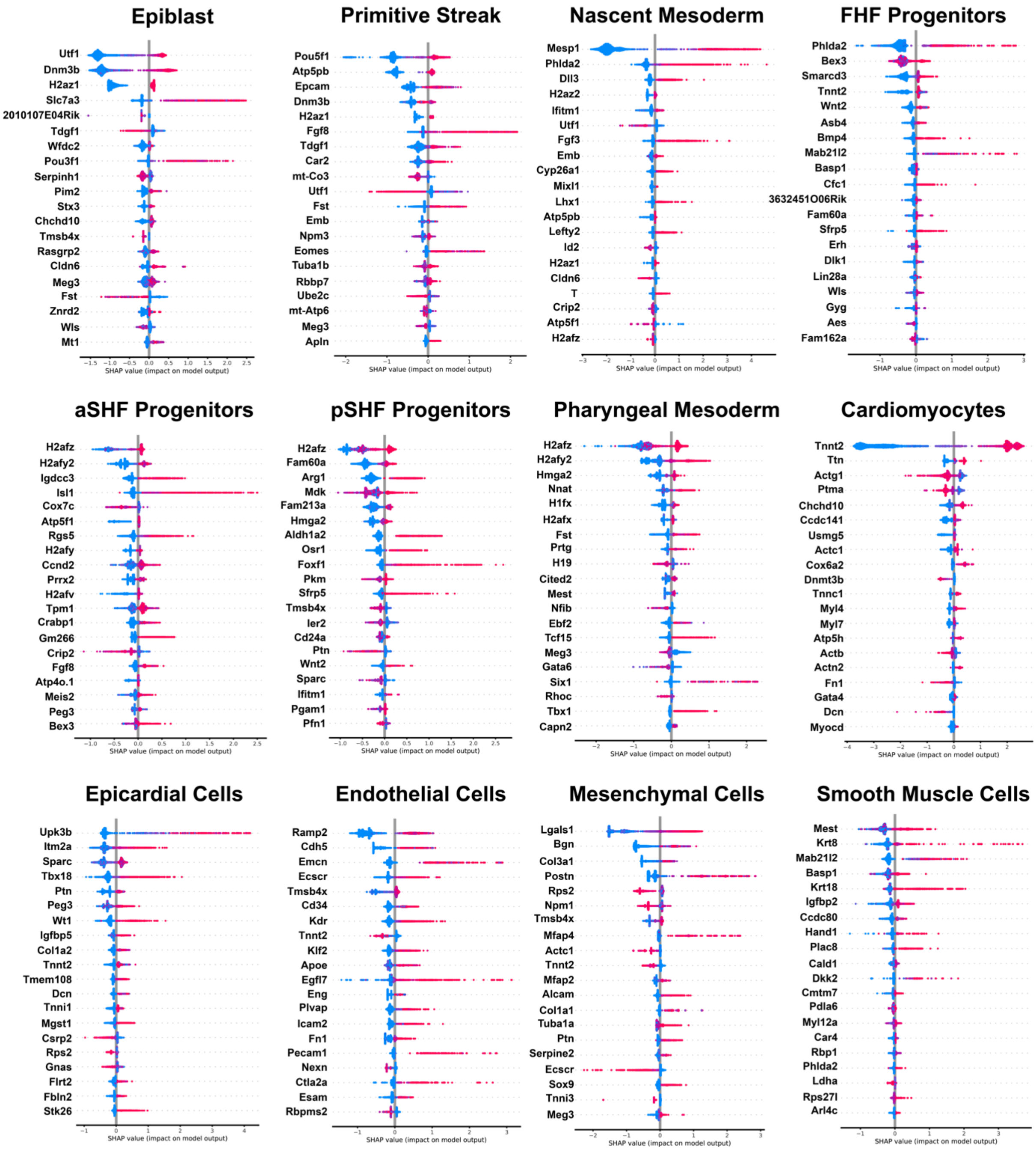

Supplementary Figure 7. Feature Ranking Plots Showing Positive and Negative Gene Predictors of Layer 1 General Cell Types. SHAP analysis was conducted on *devCellPy*'s XGBoost model trained on the general cell types (layer 1) of the cardiac developmental cell atlas. Greater SHAP values in the positive direction indicate greater weight given to a particular gene as a positive predictor of a cell identity. Conversely, highly negative SHAP values with higher feature values indicate that the gene is negative predictor of the cell identity.

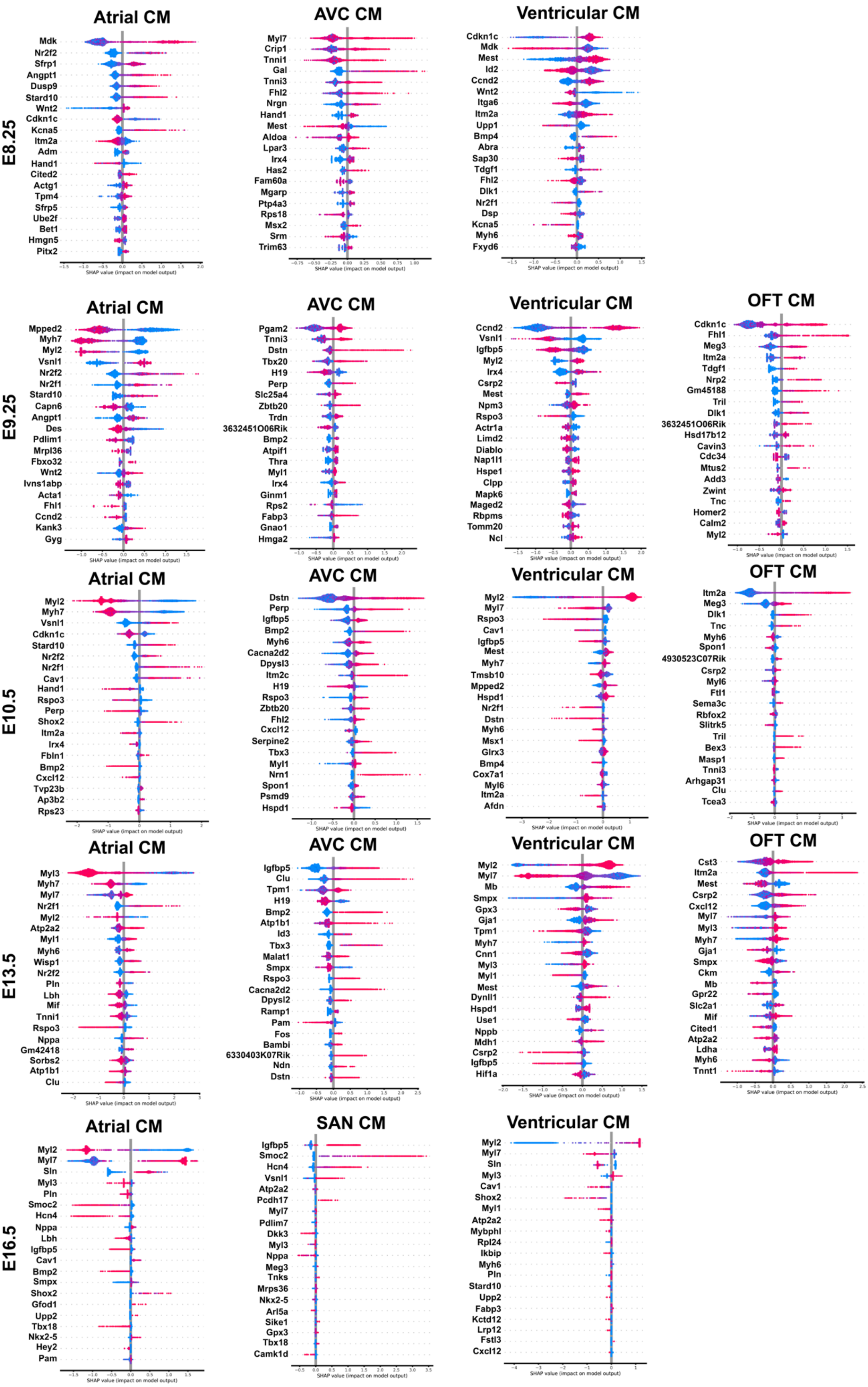

Supplementary Figure 8. Feature Ranking Plots Showing Positive and Negative

Gene Predictors of Layer 2 Cardiomyocyte Subtypes. SHAP analysis indicating top ranked positive and negative gene predictors of cardiomyocyte subtypes by timepoint model.

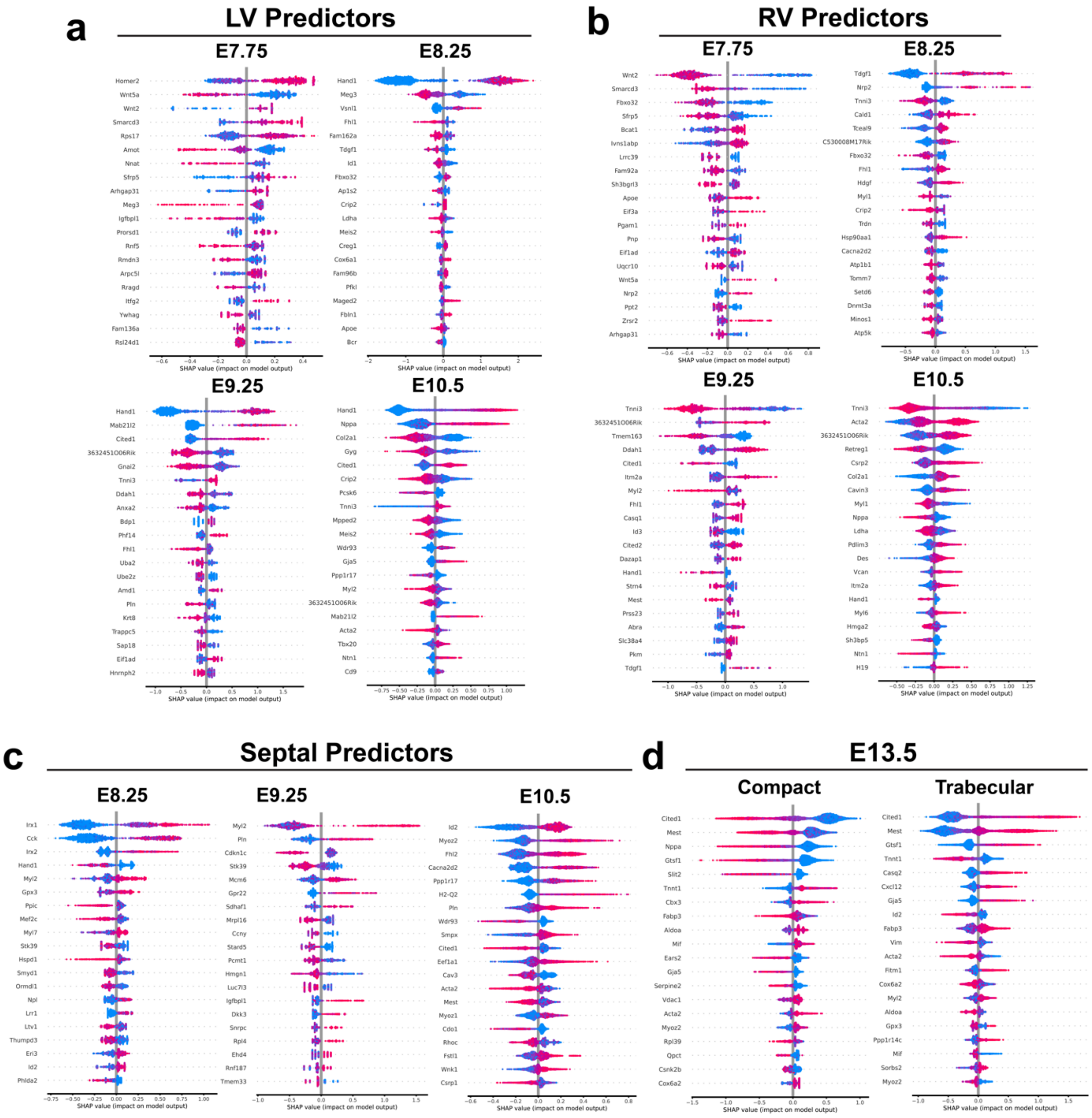

Supplementary Figure 9. Feature Ranking Plots Showing Positive and Negative Gene Predictors of Layer 3 Ventricular Cardiomyocyte Subtypes. SHAP analysis indicating top ranked positive and negative gene predictors of ventricular cardiomyocyte subtypes divided by individual timepoints. Top predictors are indicated for LV (A), RV (B), Septal (C), and E13.5 trabecular vs. trabecular cardiomyocytes (D).

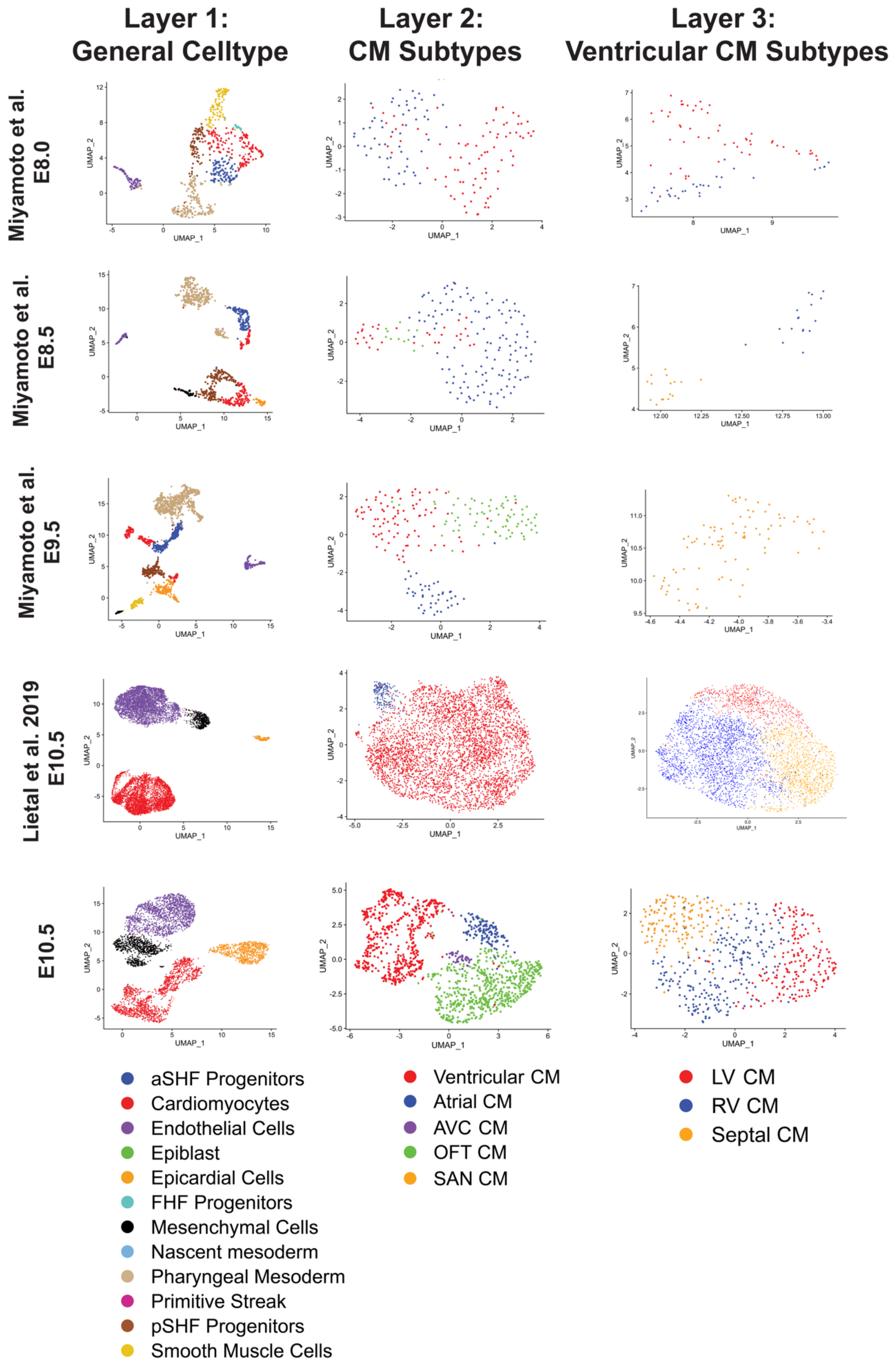

Supplementary Figure 10: Multilayered Annotation of External Datasets for *devCellPy* Testing. Datasets were downloaded for testing *devCellPy* accuracy at

predicting datasets that were independent of the cardiac developmental atlas training data. Unsupervised clustering and manual annotation were conducted across three major layers of annotation. E10.5 mouse hearts were freshly collected and annotated for further testing the *devCellPy* algorithm.

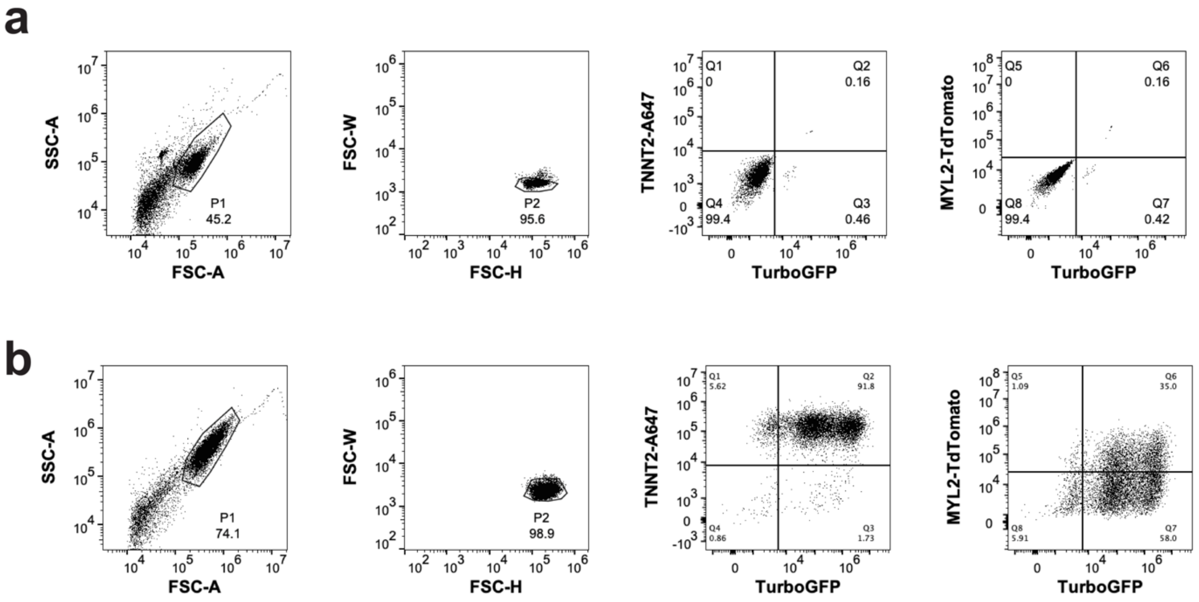

Supplementary Figure 11: Gating scheme for TBX5/MYL2 Fluorescent Reporter hiPSC-CM Cells. A) Day 3 of differentiation served as a negative control for setting gating scheme for analysis of fluorescent cell populations. **B)** Full gating scheme for flow plots reported in Figure 7C-D.

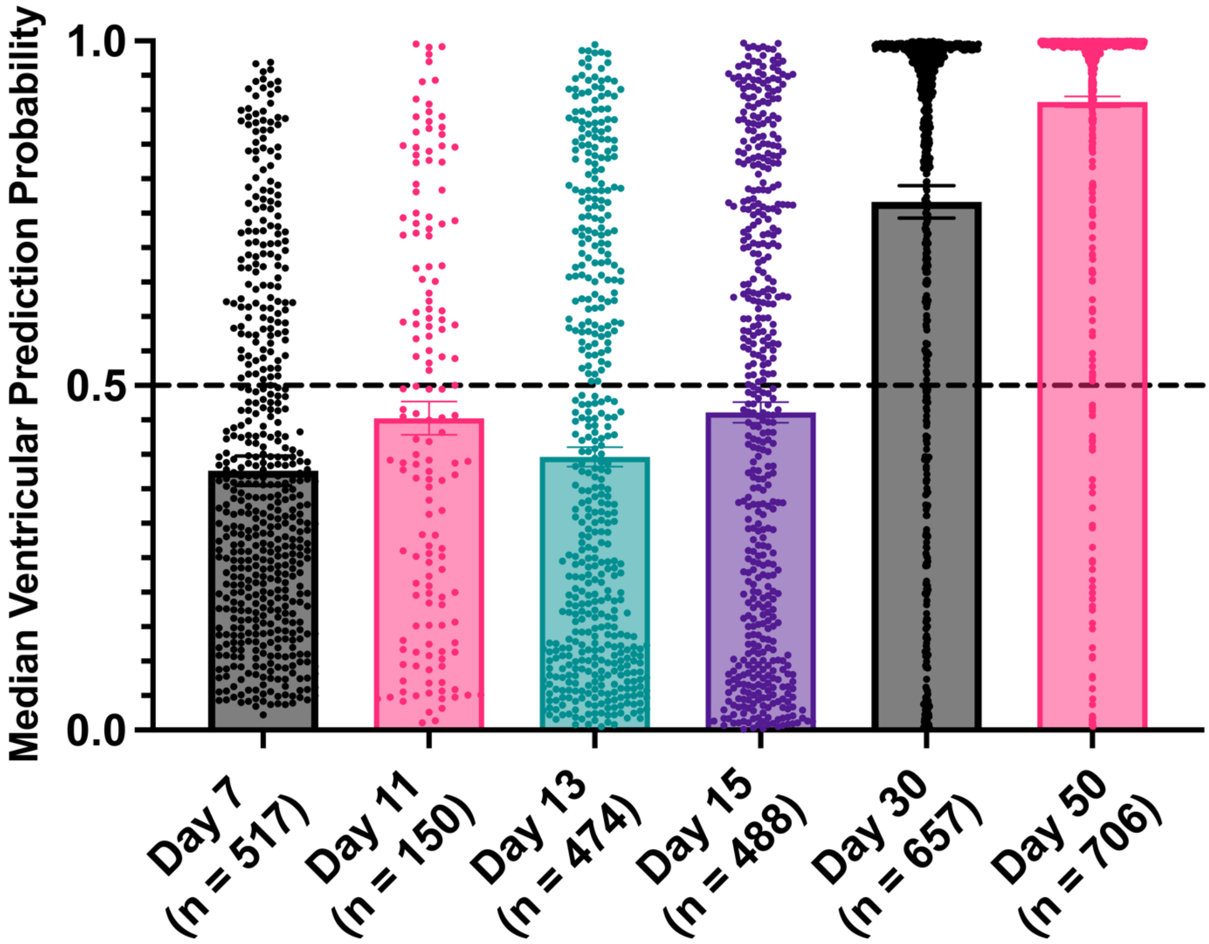

Supplementary Figure 12: *devCellPy* 5WK-7WK Human Fetal 5 Prediction Models Are Poor Predictors of Early Timepoint HiPSC Ventricular CMs. *devCellPy* was trained on 5WK-7WK Human Fetal data from Cui et al., *Cell Reports,* 2019 and applied for the prediction of hiPSC-CM time course data. Low prediction probabilities were observed for the prediction of ventricular CMs between Day 7 and 30. Error bars represent 95% confidence intervals around the median prediction probabilities. Number of cells predicted per timepoint included Day 7 = 517, Day 11 = 150, Day 13 = 474, Day 15 = 488, Day 30 = 657, Day 50 = 706.

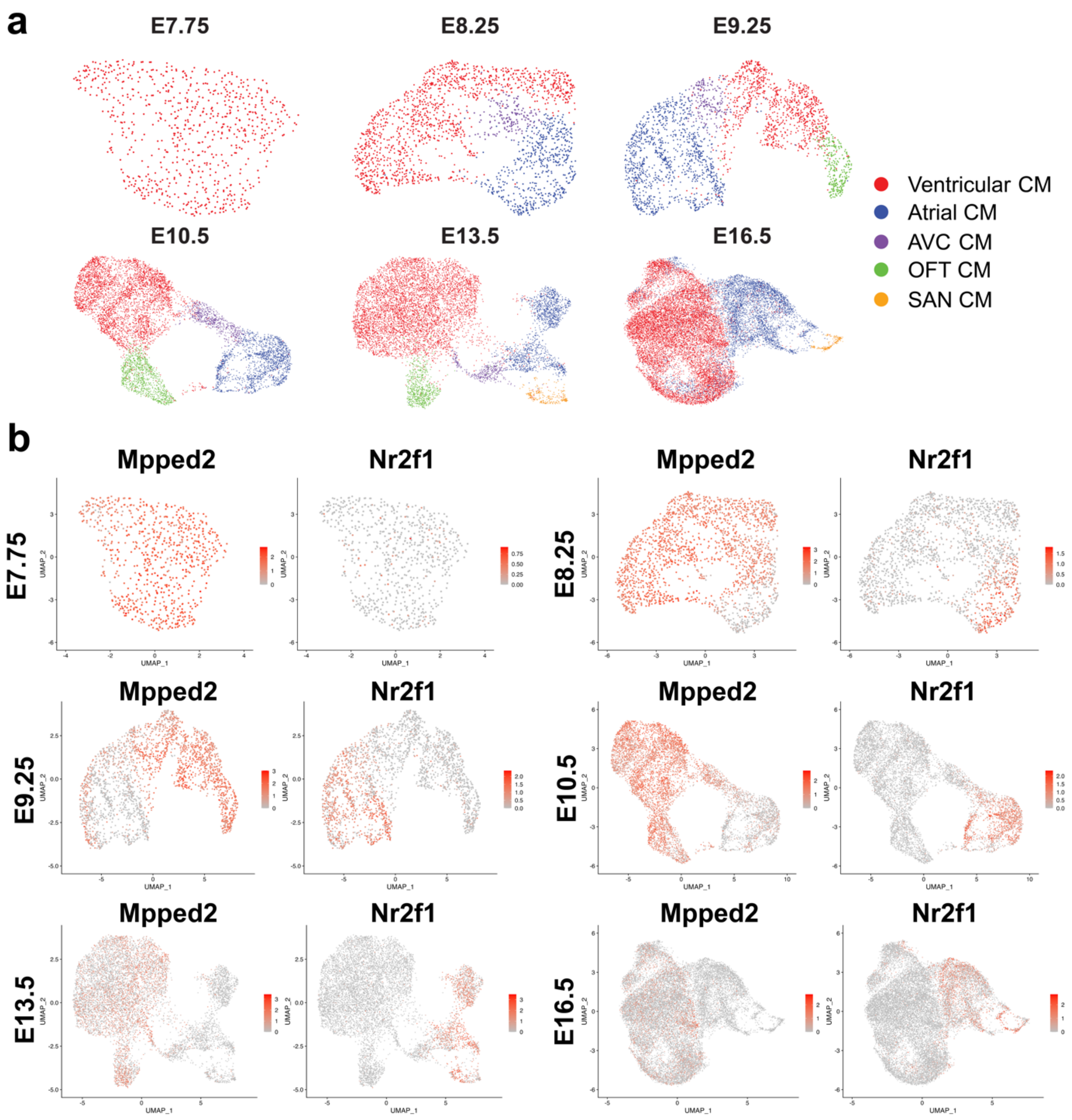

Supplementary Figure 13: Marker Expression For Annotation of Atrial and Ventricular Cardiomyocytes During Murine Cardiac Development. Additional markers, shown for the annotation of atrial and ventricular cardiomyocytes during murine development. **A)** Cluster labeling of cardiomyocyte subtypes between E7.75-E16.5. **B)** Expression of ventricular marker, *Mpped2*, and atrial marker, *Nr2f1*, across cardiac development.

Supplementary Data 1: Differential Expression Analysis for All Annotation Layers of Cardiac Developmental Atlas.

Supplementary Data 2: Performance metrics across 10-fold cross validation replicates across all layers of the cardiac atlas.

Supplementary Table 1: GEO Accessions for Published Datasets Included in Manuscript

**Reference**	**Repository**	**Accession**
Miyamoto etal. 2021	Gene Expression Omnibus	GSE165300
Pijuan-Sala etal. 2019	ArrayExpress	E-MTAB-6967
deSoysa etal. 2019	Gene Expression Omnibus	GSE126128
Hill etal. 2019	Gene Expression Omnibus	GSE131181
Goodyer etal. 2019	Gene Expression Omnibus	GSE132658
Li etal. 2019	Gene Expression Omnibus	GSE122403
Lescroart etal. 2018	Gene Expression Omnibus	GSE100471

Supplementary Table 2: GEO Accessions for human iPSC and E10.5 Mouse scRNA-seq Data

**Dataset**	**Repository**	**Accession**
SCVI-111 hiPSC-CM Time Course	Gene Expression Omnibus	GSE184943
SCVI-111 TBX5-Reporter hiPSC-CM Day 15 ICELL8 Data	Gene Expression Omnibus	GSE184943
E10.5 Mouse Heart	Gene Expression Omnibus	GSE184943
