# Supplementary material for: devCellPy is a machine learning-enabled pipeline for automated annotation of complex multilayered single-cell transcriptomic data
Source: Nat Commun. 2022 Sep 7;13:5271. doi: 10.1038/s41467-022-33045-x (PMC9452519; doi:10.1038/s41467-022-33045-x)
Supplement: Supplementary file 2 — Reporting Summary [file 41467_2022_33045_MOESM2_ESM.pdf]

nature portfolio

Corresponding author(s): Sean M. Wu
smwu@stanford.edu

Last updated by author(s): Aug 21, 2022

Reporting Summary

Nature Portfolio wishes to improve the reproducibility of the work that we publish. This form provides structure for consistency and transparency in reporting. For further information on Nature Portfolio policies, see our Editorial Policies and the Editorial Policy Checklist.

Statistics

For all statistical analyses, confirm that the following items are present in the figure legend, table legend, main text, or Methods section.

n/a	Confirmed	
☐	☒	The exact sample size (*n*) for each experimental group/condition, given as a discrete number and unit of measurement
☐	☒	A statement on whether measurements were taken from distinct samples or whether the same sample was measured repeatedly
☐	☒	The statistical test(s) used AND whether they are one- or two-sided<br>*Only common tests should be described solely by name; describe more complex techniques in the Methods section.*
☐	☒	A description of all covariates tested
☐	☒	A description of any assumptions or corrections, such as tests of normality and adjustment for multiple comparisons
☐	☒	A full description of the statistical parameters including central tendency (e.g. means) or other basic estimates (e.g. regression coefficient) AND variation (e.g. standard deviation) or associated estimates of uncertainty (e.g. confidence intervals)
☐	☒	For null hypothesis testing, the test statistic (e.g. *F*, *t*, *r*) with confidence intervals, effect sizes, degrees of freedom and *P* value noted<br>*Give P values as exact values whenever suitable.*
☒	☐	For Bayesian analysis, information on the choice of priors and Markov chain Monte Carlo settings
☐	☒	For hierarchical and complex designs, identification of the appropriate level for tests and full reporting of outcomes
☒	☐	Estimates of effect sizes (e.g. Cohen's *d*, Pearson's *r*), indicating how they were calculated

Our web collection on statistics for biologists contains articles on many of the points above.

Software and code

Policy information about availability of computer code

Data collection	Raw data for freshly collected 10X Genomics v3.1 scRNA-seq experiments (mouse 10.5 and human iPSC-CMs time course data) was aligned using the CellRanger software provided by 10X Genomics. Mouse E10.5 data and Human iPSC-CM time course data was aligned using CellRanger-v6.0.0 and further processed using the Seurat package in R (v4.1.1). Raw data for day 15 human iPSC-CM ICELL8 data was aligned using the STAR alignment software (v2.7.9a).<br><br>No software was used to collect publicly available data as the raw data was directly downloaded from public sources. For alignment of raw data from publicly available datasets we used CellRanger-v6.0.0.
Data analysis	We have deposited the code for devCellPy on GitHub (https://github.com/devCellPy-Team/devCellPy) and is available for download as a Python package in PyPi using the command "pip install devcellpy". Python package dependencies for devCellPy and full documentation can be found the devCellPy GitHub.<br><br>Preprocessing and analysis of single cell data was conducted in R using custom scripts. The following open source R packages were used for analyses and visualizations:<br>R (v4.1.1), Seurat (v4.0.5), SeuratWrappers (v4.0.2), SeuratObject (v4.0.2), SoupX (v1.5.2), celda (v1.8.1), ggplot2 (v3.3.5), ggpubr (v0.4.0), dplyr (v1.0.7), R.utils (v2.11.0), biomaRt (v2.48.3), destiny (v3.8.0), svglite (v 2.0.0), scran (v1.20.1), SingleCellExperiment (v1.14.1), scrattch.io (v0.1.0), patchwork (v1.1.1)<br><br>Flow Cytometry data was analyzed using FlowJo Version 10.8.0

For manuscripts utilizing custom algorithms or software that are central to the research but not yet described in published literature, software must be made available to editors and reviewers. We strongly encourage code deposition in a community repository (e.g. GitHub). See the Nature Portfolio guidelines for submitting code & software for further information.

Data

Policy information about availability of data

All manuscripts must include a data availability statement. This statement should provide the following information, where applicable:

- Accession codes, unique identifiers, or web links for publicly available datasets
- A description of any restrictions on data availability
- For clinical datasets or third party data, please ensure that the statement adheres to our policy

Raw scRNA-seq data for datasets that were generated for this manuscript are publicly available under the GEO accession number: GSE184943. GEO accession numbers for published datasets used in this study can be found in the Data Availability Statement of the manuscript.

Field-specific reporting

Please select the one below that is the best fit for your research. If you are not sure, read the appropriate sections before making your selection.

☒ Life sciences ☐ Behavioural & social sciences ☐ Ecological, evolutionary & environmental sciences

For a reference copy of the document with all sections, see nature.com/documents/nr-reporting-summary-flat.pdf

Life sciences study design

All studies must disclose on these points even when the disclosure is negative.

Sample size	Sample sizes for testing the accuracy of devCellPy algorithm for the prediction of cell identities were determined by conducting an analysis of the minimum number of cell numbers necessary to conduct accurate cell predictions. Analysis revealed that a minimum of 16 cells per cell category for training the algorithm provided an average accuracy 83% and a minimum of 128 cells per category leading to 95% accuracy in prediction. Given this, we determined that greater than 32 cells was a sufficient sample size for training the algorithm and evaluation of its overall accuracy. For testing devCellPy, a 10-fold cross validation was implemented whereby a test data set was randomly divided into stratified partitions of 90 and 10 percent such that the 90% partition was used for training and the 10% was used for evaluation of the algorithm. By conducting 10-fold random shuffling and stratified repartitioning, the entire dataset could be tested in individual folds and a statistical calculation of devCellPy's overall prediction error could be calculated.<br><br>All single cell RNA-seq datasets used in this study containing cells on the order of thousands of cells with multiple hundred of cells per cell type assessed in all experiments. Given this number, this provided sufficient cell numbers (>32 cells) per cell category necessary for training devCellPy. DevCellPy does not have a minimum number required for cell prediction and can predict as little as 1 cell, thus allowing for the evaluation of the algorithm on smaller datasets that the algorithm has never encountered.<br><br>For flow cytometry experiment using TBX5 reporter cell line (Figure 8D), experiments were conducted with 23 independent biological replicates which vastly exceeds the minimum requirement of 3 biological replicates to conduct statistical calculations of standard error.
Data exclusions	For the public datasets utilized in the construction of a cardiac developmental cell atlas we filtered out low quality cells by excluding cells where the number of genes, number of mRNA counts, percent mitochondrial gene expression, or percent ribosomal gene expression exceeded the median of these metrics by plus or minus three times the median absolute deviation. Given the focus of our study was on mesodermal derivative during cardiac development we filtered non-mesodermal cell types which included endodermal and ectodermal germ layer derivatives that had been previously identified in the original papers from which the data was obtained. For datasets used for testing the devCellPy algorithm including datasets from Lescroart etal 2019, Li etal 2019, Miyamoto etal 2021 we followed the same procedure and excluded non-mesodermal cell types in order to test the concordance of devCellPy's cell predictions with manual annotation of unsupervised clusters.<br><br>For freshly collected datasets in this manuscript including the E10.5 mouse embryonic heart data and human iPSC-derived cardiomyocyte data collected, we again filtered out low quality cells by excluding cells where the number of genes, number of mRNA counts, percent mitochondrial gene expression, or percent ribosomal gene expression exceeded the median of these metrics by plus or minus three times the median absolute deviation. For the mouse E10.5 data, we filtered non-mesodermal cell types for testing of the devCellPy-generated cardiac prediction algorithm. For the human iPSC-derived cardiomyocyte data, we focused our analysis on cardiomyocyte populations and therefore filtered out non-cardiomyocyte populations based on the expression of established canonical cardiomyocyte markers TNNT2, ACTA2, and TNNI1.
Replication	We demonstrate the high performance of the devCellPy prediction pipeline using multiple datasets in our manuscript. The first large dataset that we used for testing devCellPy was a cardiac atlas consisting of greater than 100,000 cells. To test the performance of the algorithm on each layer of annotation, each layer was trained independently on subsets of the scRNA-Seq atlas. For each layer tested, the data was divided into a 90% partition for cross validation and a 10% hold-out dataset. Subsequently, the 90% cross-validation partition underwent further partitioning into a 90% segment used for training and a 10% segment used for calculation of performance metrics. 10-fold cross validation was conducted by randomly reshuffling the data used for training and testing to obtain a statistical estimate of the model's error for its overall accuracy, precision, recall, and F1-scores. After 10-fold cross validation, we fed the 10% held-out partition from the initial subdivision and calculated the confusion matrices to determine the prediction accuracy of the algorithm across all cell classes.<br><br>To further test the devCellPy-generated cardiac prediction models on data that the algorithm had not previously seen, , independent murine cardiac cell datasets were downloaded from Lescroart et al 2018, Miyamoto et al 2021, and Li et al 2019. Following the same procedure as

that used for the cardiac developmental cell atlas we conducted quality control, normalization, and a multilayered manual annotation of cell types. Moreover, we collected E10.5 mouse hearts and conducted scRNA-seq on these hearts to obtain additional data for testing devCellPy on never-before-seen data. We followed the same analysis procedure as the cardiac developmental cell atlas for manual annotation of distinct cell types. Normalized gene expression matrices were exported for each of these datasets and were input into devCellPy. To compare manual annotations to devCellPy predictions for each layer of annotation, we fed devCellPy each layer individually based on the manual annotations assigned during unsupervised clustering. We calculated confusion matrices to compare the manual label versus the devCellPy predictions for cells that were successfully classified.

We conducted additional tests to show that devCellPy accuracy at cell predictions could be replicated with cross species prediction. To do this we applied the algorithm on human iPSC-derived cardiomyocytes and correlated the predictions with the results of a LV-specific lineage tracing system. We show that the algorithm agreed with the lineage tracing data therefore showing a further level of replication.

Randomization: NA. This study did not require experimental grouping.

Blinding: NA. This study did not require experimental grouping or blinding.

Reporting for specific materials, systems and methods

We require information from authors about some types of materials, experimental systems and methods used in many studies. Here, indicate whether each material, system or method listed is relevant to your study. If you are not sure if a list item applies to your research, read the appropriate section before selecting a response.

Materials & experimental systems

n/a	Involved in the study
☐	☒ Antibodies
☐	☒ Eukaryotic cell lines
☒	☐ Palaeontology and archaeology
☐	☒ Animals and other organisms
☒	☐ Human research participants
☒	☐ Clinical data
☒	☐ Dual use research of concern

Methods

n/a	Involved in the study
☒	☐ ChIP-seq
☐	☒ Flow cytometry
☒	☐ MRI-based neuroimaging

Antibodies

Antibodies used: Cardiac Troponin T monoclonal antibody (clone 13-11) was used for flow cytometry (ThermoFisher Catalog Number: MA5-12960). Secondary antibody used in this study was a goat anti-mouse IgG Alexa Fluor 647 antibody (Thermofisher Catalog Number: A-21235)

Validation: Cardiac Troponin T antibody used in this study is a well referenced and validated antibody used in the field of cardiac biology. The antibody has undergone advanced verification by the manufacturer by relative expression to ensure the antibody binds to the antigen as stated (refer to manufacturer statement: https://www.thermofisher.com/antibody/product/Cardiac-Troponin-T-Antibody-clone-13-11-Monoclonal/MA5-12960). Secondary antibody used in this study has also been well cited as referenced by the manufacturers website (https://www.thermofisher.com/antibody/product/Goat-anti-Mouse-IgG-H-L-Cross-Adsorbed-Secondary-Antibody-Polyclonal/A-21235)

Eukaryotic cell lines

Policy information about cell lines

Cell line source(s): Human Induced Pluripotent Pluripotent Stem Cell Line SCVI-111 obtained from the Stanford Cardiovascular Institute Biobank

Authentication: Line generated and authenticated by Stanford Cardiovascular Institute Biobank. SNP analysis performed to match iPSC lines to original patient cells and karyotypic performed for each line.

Mycoplasma contamination: All cell lines tested negative for mycoplasma contamination.

Commonly misidentified lines (See ICLAC register): No misidentified lines were used.

Animals and other organisms

Policy information about studies involving animals; ARRIVE guidelines recommended for reporting animal research

Laboratory animals: Mouse strain used for all experiment were embryonic day 10.5 CD1 wildtype mix of male and female embryos. Time pregnancies obtained commercially by Jackson Laboratories and embryos were collected upon arrival of animals to laboratory.

Wild animals: Study did not involve wild animals.

Field-collected samples: Study did not involve field-collected samples.

Ethics oversight | All animal experiments described have been approved by the Administrative Panel on Laboratory Animal Care at Stanford University.

Note that full information on the approval of the study protocol must also be provided in the manuscript.

Flow Cytometry

Plots

Confirm that:

- [x] The axis labels state the marker and fluorochrome used (e.g. CD4-FITC).
- [x] The axis scales are clearly visible. Include numbers along axes only for bottom left plot of group (a 'group' is an analysis of identical markers).
- [x] All plots are contour plots with outliers or pseudocolor plots.
- [x] A numerical value for number of cells or percentage (with statistics) is provided.

Methodology

Sample preparation

A Beckman Coulter CytoFLEX flow cytometer was used for high throughput analysis of TNNT2, TurboGFP, and MYL2-TdTomato expression of hiPSC-CM derived from genome edited hiPSC containing the TBX5-lineage tracing reporter system. On days 3 and 35 of collection, cells were dissociated to single cells in 10X TrypLE Select (ThermoFisher) for 5 minutes at 37 degrees Celsius. Cells were subsequently pelleted by centrifugation at 200g for 5 minutes. Cell pellets were resuspended in 4% PFA for 10 minutes and were rinsed with a 5% Fetal Bovine Serum solution in 1x PBS. Cells were permeabilized in a 0.5% Saponin solution containing 5% FBS in 1X PBS (hereafter refered to as Saponin Solution). After permeabilization cells were incubated for 45 minutes in a monoclonal mouse anti-Troponin primary antibody (ThermoFisher Cat. MA5-12960) at a 1:100 dilution in 0.5% saponin solution. Cells were rinsed twice in saponin solution and then incubated in secondary antibody AlexaFluor 647 goat anti-mouse (ThermoFisher) at a 1:1000 dilution in 0.5% saponin solution. Cells were subsequently rinsed in 1x PBS twice and analyzed using CytoFLEX flow cytometer. Flow cytometry data analyzed with FlowJo analysis software version 10.8.0. 23 independent biological replicates were analyzed for analysis of GFP expression and MYL2-TdTomato expression in hiPSC-derived cardiomyocytes as reported in Figure 7 and Supplementary Figure 8. Gating scheme was set relative to day 3 of differentiation which served as the control for analyzing fluorescent cell populations.

Instrument

Beckman Coulter CytoFLEX Flow Cytometer

Software

FlowJo version 10.8.0

Cell population abundance

Relevant cell populations analyzed included the percent TurboGFP-postive cells out of the TNNT2-A647-positive population. This population represents the percentage of cardiomyocytes that exhibit expression of TurboGFP. Across 23 independent differentiations, we calculated an average GFP+/TNNT2+ population of 81.3% with a standard error of the mean of 3.0%. The second relevant population analyzed was the percent GFP+/MYL2-TdTomato+ population which indicates the number of GFP + cells among ventricular cardiomyocytes expressing the MYL2-TdTomato marker.

Gating strategy

Day 3 of hiPSC cardiac differentiation was used as the baseline sample for setting all gates in the flow cytometry experiment. For each sample, FSC/SSC gates were determine based on the clustering of singlet cells and for exclusion of debris. Single cell populations were subsequently carried forward to set quad gates in plots analyzing TNNT2-A647 vs TurboGFP and MYL2-TdTomato vs. TurboGFP. Day 3 cells served a negative control for TNNT2-A647, MYL2-TdTomato, and TurboGFP markers analyzed thus allowing for the determination of gating for determination of fluorescent marker expression.

- [x] Tick this box to confirm that a figure exemplifying the gating strategy is provided in the Supplementary Information.
