# Supplementary material for: devCellPy is a machine learning-enabled pipeline for automated annotation of complex multilayered single-cell transcriptomic data
Source: Nat Commun. 2022 Sep 7;13:5271. doi: 10.1038/s41467-022-33045-x (PMC9452519; doi:10.1038/s41467-022-33045-x)
Supplement: Supplementary file 3 — Description of Additional Supplementary Files [file 41467_2022_33045_MOESM3_ESM.pdf]

Title: Supplementary Data 1:
Description: Differential Expression Analysis for All Annotation Layers of Cardiac Developmental Atlas.

Title: Supplementary Data 2:
Description: Performance metrics across 10-fold cross validation replicates across all layers of the cardiac atlas.
